# Unsupervised Machine Learning on Motion Capture Data Uncovers Movement Strategies in Low Back Pain

**DOI:** 10.3389/fbioe.2022.868684

**Published:** 2022-04-14

**Authors:** Anastasia V. Keller, Abel Torres-Espin, Thomas A. Peterson, Jacqueline Booker, Conor O’Neill, Jeffrey C Lotz, Jeannie F Bailey, Adam R. Ferguson, Robert P. Matthew

**Affiliations:** ^1^ Brain and Spinal Injury Center (BASIC), Weill Institute for Neuroscience, Department of Neurological Surgery, University of California, San Francisco, San Francisco, CA, United States; ^2^ San Francisco Veterans Affairs Healthcare System, San Francisco, CA, United States; ^3^ Department of Orthopaedic Surgery, University of California, San Francisco, San Francisco, CA, United States; ^4^ Department of Physical Therapy and Rehabilitation Science, University of California, San Francisco, San Francisco, CA, United States

**Keywords:** nonlinear principal component analysis, biomechanics, chronic low back pain, sit-to-stand, movement strategies

## Abstract

Chronic low back pain (LBP) is a leading cause of disability and opioid prescriptions worldwide, representing a significant medical and socioeconomic problem. Clinical heterogeneity of LBP limits accurate diagnosis and precise treatment planning, culminating in poor patient outcomes. A current priority of LBP research is the development of objective, multidimensional assessment tools that subgroup LBP patients based on neurobiological pain mechanisms, to facilitate matching patients with the optimal therapies. Using unsupervised machine learning on full body biomechanics, including kinematics, dynamics, and muscle forces, captured with a marker-less depth camera, this study identified a forward-leaning sit-to-stand strategy (STS) as a discriminating movement biomarker for LBP subjects. A forward-leaning STS strategy, as opposed to a vertical rise strategy seen in the control participants, is less efficient and results in increased spinal loads. Inefficient STS with the subsequent higher spinal loading may be a biomarker of poor motor control in LBP patients as well as a potential source of the ongoing symptomology.

## Introduction

Low back pain (LBP) is a leading cause of disability and opioid prescriptions worldwide (Ringwalt et al., 2014; Vos et al., 2016) with significant medical and socioeconomic impact estimated at $87 billion (Dieleman et al., 2016) per year in healthcare spending. Specific pathologies such as cancer, fracture, and infection account for less than 6% of cases, with the vast majority of LBP cases classified as non-specific LBP (Henschke et al., 2009; Hartvigsen et al., 2018). The lack of clinical tests to identify relevant biopsychosocial processes responsible for an individual’s LBP experience has resulted in overall poor patient outcomes, despite extensive research efforts (Woolf et al., 1998; Burton, 2005; von Hehn et al., 2012; Hush et al., 2013; Hodges, 2019). A current priority of LBP research is the development of widely adoptable quantitative assessments to classify individual patients and select appropriately targeted therapies (Woolf et al., 1998; Foster et al., 2009; Costa et al., 2013; Hush et al., 2013).

Low back pain is associated with altered sensorimotor processes (Hodges, 2000; Hodges and Smeets, 2015; Hodges et al., 2019; van Dieën et al., 2019). Nociceptive input modulates sensorimotor function by altering spinal circuits of the central nervous system (CNS) (Schomburg et al., 2001; Amann et al., 2013; Mandadi et al., 2013; Sidhu et al., 2017; Massé-Alarie et al., 2019). The intrinsic interaction between pain processing and sensorimotor circuitry provides a physiological basis for biomechanical biomarkers as a functional readout of the CNS response to LBP (Amann et al., 2013; Sidhu et al., 2017; Massé-Alarie et al., 2019). Using kinematics and electromyography, prior work demonstrated distinct neuromuscular mechanics of LBP patients compared to healthy controls (Shum et al., 2005a; Shum et al., 2005b; Moseley and Hodges, 2006; Shum et al., 2007a; Shum et al., 2007b; Christe et al., 2016; Papi et al., 2018). Kinematic measures, such as position, velocity and acceleration, and particularly their interaction (acceleration at a specific velocity as a patient moves through a trunk-specific angle) can predict LBP etiology with 70% accuracy. In contrast, routine medical imaging techniques aiming to identify the anatomic source of LBP stratify patients by etiology in less than 20% of the cases (Marras et al., 1995, 19). Together, these suggest that high-fidelity analysis of movement has the potential to serve as a diagnostic biomarker of LBP that rivals medical imaging in diagnostic accuracy. The addition of accurate, movement biomarkers has the potential to improve patient care by identifying sub-populations who may be strong responders to particular therapies, or identify those who may be at risk. To date, motion-based studies in LBP populations have mainly assessed trunk kinematics and/or kinetics while patients performed isolated movements (e.g., bending, sitting, or standing). In this study, we assessed the coordinated movement of the spine and lower limbs using the sit-to-stand (STS) maneuver. The five-times STS or 30 second STS tests are commonly used clinical assessments of lower extremity strength and have been used to assess if a person can independently perform activities of daily living (Rolfson et al., 2006). The movement requires coordinated effort between the lumbar spine, hip, and knees (Shum et al., 2005a), and has been used to elicit compensatory movements in different clinical populations including those with low back pain (Ippersiel et al., 2018; Staartjes and Schröder, 2018).

A challenge for point-of-care biomechanical assessment is its dependency on complex measurement systems developed for research settings that typically require instrumenting the patient. High-accuracy research systems are incompatible with routine use in the clinic because of the cost, space, time, and expertise requirements needed to conduct conventional motion assessments. Alternatively, marker-less motion capture using standard color and depth cameras has been used to collect movement data in clinic. One of these systems utilizing the Microsoft Kinect cameras has been validated for kinematic and dynamic measures during gait (Pfister et al., 2014), standing balance (Clark et al., 2012), and STS (Matthew et al., 2019b; Matthew et al., 2019a).

In addition to implementation challenges associated with accurately collecting raw movement data, is the identification of clinically relevant summary metrics. To address this, we performed integrative analysis using unsupervised machine learning from marker-less STS data collected on a convenience sample of three subject types: 1) Non-specific LBP patients; 2) patients with LBP due to spinal deformity, and 3) healthy controls. We hypothesized that unsupervised machine learning on full body biomechanics would identify divergent movement patterns that discriminate LBP patients from healthy controls. Our analysis unveils quantitative STS strategies that describe clinically relevant movement patterns.

## Materials and Methods

### Participants

This study consists of three cohorts: individuals with non-specific low back pain (NS-LBP, n = 43), individuals who are considering spinal fusion due to adult spinal deformity (SD-LBP; n = 42), and controls (n = 26) (see Table 1 for details on demographics). Patients were recruited during scheduled appointments for low back pain or pre-surgical consultation due to degenerative spinal conditions that result in thoracolumbar malalignment. The control cohort was recruited from the clinicians, staff, and students at the recruitment site. This research study was conducted in accordance with the Declaration of the World Medical Association. All subjects signed the informed consent (UCSF IRB: 16-21015, 17-22291).

**TABLE 1:** Participant demographics.

	Back pain(N = 43)	Control(N = 26)	Surgery(N = 42)	Total(N = 111)	F/chi-square*p*value
Age					
Mean (SD)	53.9 (17.4)	27.5 (8.85)	62.7 (11.9)	51.0 (19.3)	F (2, 108) = 54.13
Median [min, max]	54.0 [21.0, 85.0]	24.0 [18.0, 58.0]	64.5 [30.0, 80.0]	55.0 [18.0, 85.0]	*p* <0.0001*
Sex					
Mean (SD)	0.558 (0.502)	0.500 (0.510)	0.310 (0.468)	0.450 (0.500)	X^2^ (2) = 5.64
Median [min, max]	1.00 [0, 1.00]	0.500 [0, 1.00]	0 [0, 1.00]	0 [0, 1.00]	*p* =0.059
BMI					
Mean (SD)	26.7 (4.18)	23.8 (3.85)	26.4 (4.86)	25.9 (4.49)	F (2, 108) = 3.82
Median [min, max]	27.0 [20.0, 41.0]	23.0 [18.0, 33.0]	27.0 [17.0, 38.0]	26.0 [17.0, 41.0]	*p* =0.024**
VAS					
Mean (SD)	4.67 (2.38)	NA	6.83 (2.93)	5.65 (2.84)	F (2, 108) = 12.81
Median [min, max]	5.00 [0, 9.00]	NA	8.00 [1.00, 10.0]	6.00 [0, 10.0]	*p* <0.001***
Missing	1 (2.3%)	26 (100%)	7 (16.7%)	34 (30.6%)	
ODI					
Mean (SD)	50.2 (16.3)	NA	48.9 (16.2)	49.6 (16.2)	F (2, 108) = 0.12
Median [min, max]	48.0 [20.0, 88.0]	NA	52.0 [8.00, 78.0]	50.0 [8.00, 88.0]	*p* = 0.073
Missing	2 (4.7%)	26 (100%)	9 (21.4%)	37 (33.3%)	

BMI, body mass index; VAS, Visual Analogue Scale; ODI, Oswestry Disability Index; SD, standard deviation; sex coded as 0 = female, 1 = male; Tukey post hoc results: *control vs. NS-LBP (*p* <0.0001), control vs. SD-LBP (*p* <0.0001), NS-LBP, vs. SD-LBP (*p* =0.01); **control vs. NS-LBP (*p* = 0.028), control vs. SD-LBP (*p* =0.051), NS-LBP, vs. SD-LBP (*p* =0.96); ***NS-LBP, vs. SD-LBP (*p* <0.001).

### Experimental Procedures

Patient movements were collected in the clinic before or after their regularly scheduled appointments. Subjects were provided an armless chair 17.75 inches high, with a Kinect 2 (Microsoft, Redmond, WA, United States) depth camera placed 84 inches in front of them, 45 inches off the ground. Subjects were told to place their feet so that their shanks were normal to the ground. Subjects were asked to perform three self-paced sit-to-stand actions keeping their arms by their side. Subjects were allowed to perform less than three actions if they moved their feet, used their arms, or if the investigator or subject wanted to stop the experiment.

### Data Processing

The Kinect body tracking library (C++) was used to obtain estimates of body pose from the color and depth data (Zhang, 2012). Estimates of joint position were streamed into a log file using custom software for post-processing at 30 Hz. The complete modelling approach used in this study is covered in previous work (Matthew et al., 2019b; Matthew et al., 2019a). A quadruple-pendulum planar model was used to model the movements of the ankle, knee, hip, and L5S1 joints. Segment geometry and inertia were scaled based on the subject sex, height, and mass using the relationships from (Mcconville et al., 1980; Young et al., 1983; Chaffin et al., 2006; Dumas et al., 2007). Inverse kinematics was performed using an unscented Kalman filter to obtain smoothed estimates of joint position and the corresponding joint angle (Julier and Uhlmann, 1997). Angular derivatives were obtained using sequential second-order, low-pass Butterworth filtering at 5 Hz, and numerical differentiation.

The filtered kinematic time series data were used to estimate the forces, and torques at each joint were computed using the inverse dynamics formulation by Park et al. (1995). The world-frame body segment positions, velocities, and accelerations were computed in a forward recursion from the computed joint angles, velocities, and accelerations. The contact forces and torques at each joint were then computed in a backward recursion, using the inertial parameters from the allometrically scaled planar pendulum model. Estimated joint powers were calculated from these torques and the corresponding joint angular velocities. Numerical integration was used to compute the concentric and eccentric work for each joint (Zhang et al., 2000). A planar muscular model was used to find the total loading at the L5S1 joint using the approach from Chaffin et al. (2006). This model estimates the effective muscle force and abdominal pressure from the computed torques and angles at the L5S1 joint. All measures that are derived from this analysis are normalized by the height and mass of the subject using the convention proposed by Pinzone et al. (2016). From our prior validation studies, this approach has been shown to have an average joint position error of 1.80 cm compared to active motion capture, and 30N average error compared to floor-mounted force platforms (Matthew et al., 2019a; Matthew et al., 2019b). The angle and torque time series data from the Kinect system were found to be concordant (0.81–0.99, 0.89–0.97) compared to our baseline active motion capture system.

### Measured and Derived Biomechanical Variables

The biomechanical set of variables included trunk and lower limb positions and angles (L5S1, hip, knee, and ankle angles measured with two different reference points: joint and world), velocities and accelerations (anterior and vertical velocities and accelerations calculated for each joint and body segment (torso, pelvis, thigh, and shank) with three different reference points: joint, body and world), dynamics (torques and powers calculated around each joint and normalized to patient’s height and weight) as well as compressive, shear and maximum muscle forces calculated at the L5S1 joint. The maximum and minimum variants of joint angles, accelerations, velocities, torques and powers, and maximum muscle forces around L5S1 were compiled for the data analysis. There were a total of 118 biomechanical derived variables included in the analysis. (see Data Dictionary, in Supplementary Material S1 (pages 1–10), which has a full description of each variable).

### Analytical Workflow and Statistics

**FIGURE 1:**
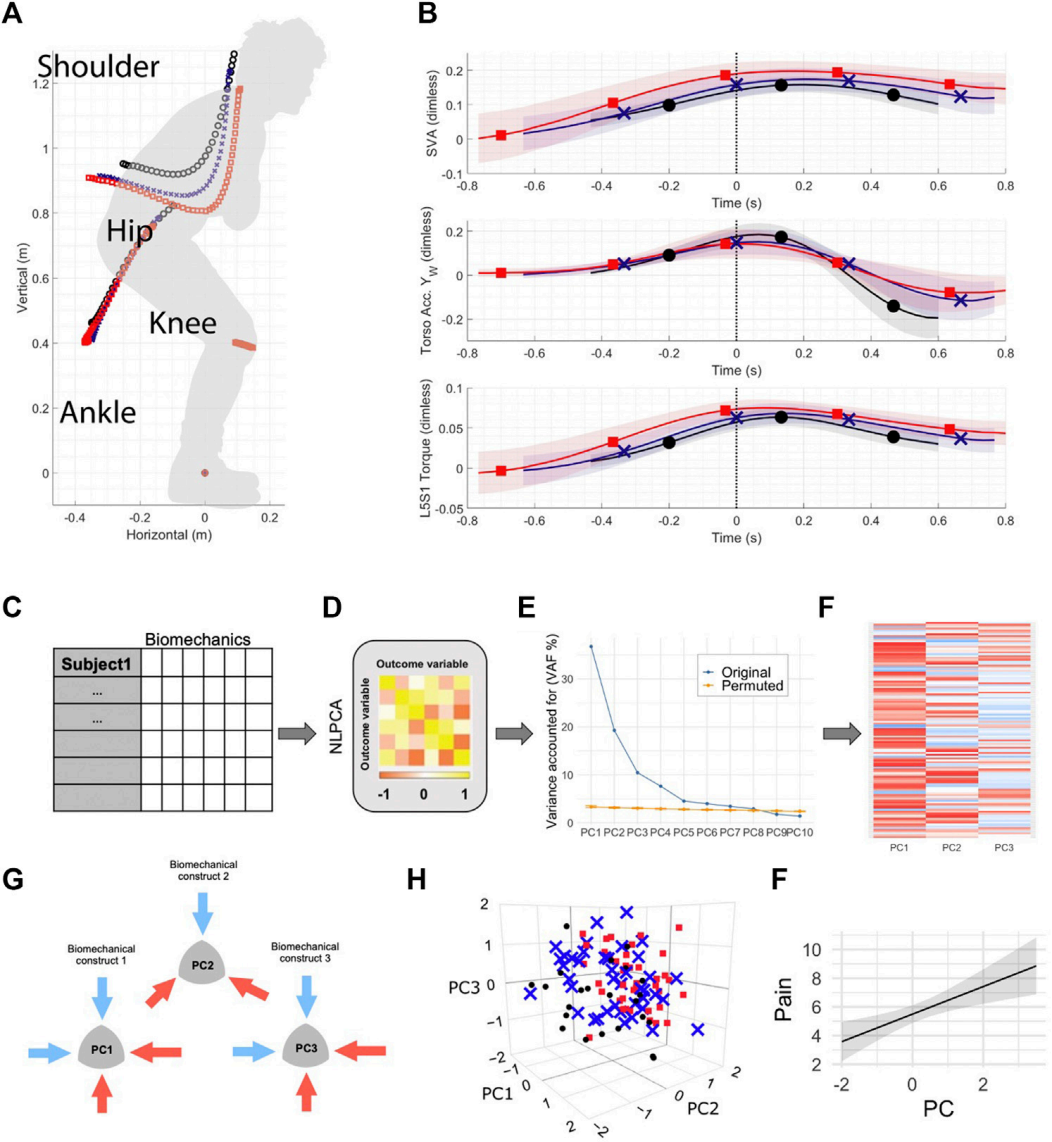
Unsupervised machine learning on full body biomechanics analytical workflow. **(A)** Healthy control (n = 23), patients with non-specific low back pain (n = 43) and patients with spinal deformity (n = 42) were assessed using full body biomechanics (59 variables) during sit-to-stand movement. **(B)** Full time series data were summarized into minimum and maximum for each variable (total of 118 variables) for each patient. Here, the time series trajectories represent group mean and standard deviation (black ∼ control, blue ∼ non-specific low back pain patients and red ∼ patients with spinal deformity) for one of the variables with a significant loading in PC1, PC2 or PC3. **(C)** The resulting dataset was analyzed using nonlinear principal component analysis **(D)**, followed by a permutation test **(E)** that determined the number of principal components (PCs) to be retained with a significant number of loadings above 0.5 **(F)**. **(G)** The first three PCs were assessed for construct validity, with subsequent generation of the biomechanical constructs of movement patterns/strategies. **(H)** Color-coded visualization of groups projected in 3D space revealed group separation particularly across PC2. **(I)** Linear regression models were then used to test whether identified movement constructs in PC1, PC2, and PC3 could predict patient-reported outcomes of pain and disability.

Figure 1 shows the schematic of the overall analytical workflow. The statistical analysis was performed using R (R: The R Project for Statistical Computing, R Project, 2021) and syndromic R package (Torres-Espín et al., 2021) specifically developed to facilitate post hoc principal component analysis (PCA), interpretation, and visualization of the PCA solutions. For the analysis, the biomechanics data were averaged across trials and repetitions of sit-to-stand as PCA suffers from intra-subject correlation and assumes independence of observations. Several studies on the kinematics of human movement comparing linear and nonlinear analytical methodologies have demonstrated that nonlinear methods outperform linear in the percent variance accounted for in the data (Harbourne et al., 2009; Portnova-Fahreeva et al., 2020; Boe et al., 2021). Furthermore, if the variables are linearly related, then PCA and NLPCA both result in the same solution. Therefore, to consider the likelihood of nonlinear relationships between the large number of biomechanical variables in our data, we have chosen to use a nonlinear approach.

**FIGURE 2:**
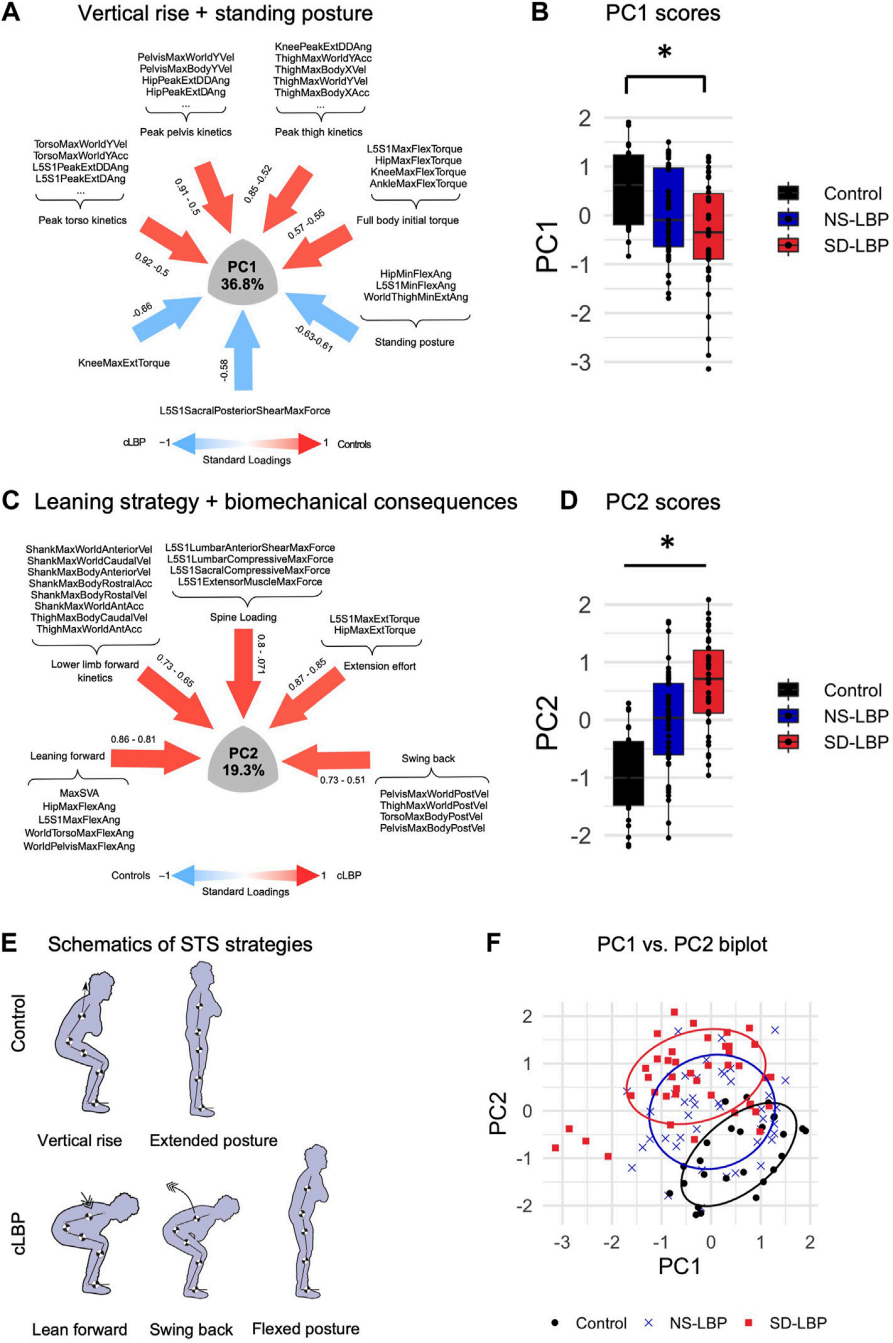
NLPCA on full body biomechanics separates across two patient subgroups and healthy controls based on STS strategies. **(A)** SyndRomics plot of PC1 loadings. Kinetic variables with highest loadings were grouped based on body segment to identify the underlying construct of vertical rise strategy. **(B)** Boxplot of PC1 scores group data. Controls had significantly higher PC1 scores compared to SD-LBP group. **(C)** SyndRomics plot of PC2 loadings. The variables were grouped based on their biomechanical identity (kinetic, kinematic, dynamics or muscle forces) revealing the underlying construct of leaning sit-to-stand strategy that results in increased spinal loading requiring a more laborious extension effort in patients with LBP vs. controls. **(D)** Boxplot of PC2 scores group data. There was a significant difference between all three groups. **(E)** Schematic representation of the control and LBP patient sit-to-stand (STS) strategies captured in PC1 and PC2. F. PC1 vs. PC2 biplot visualizes the group separation in 2D space. *Significance established at *p* ≥ 0.5; ANOVA on PC1 scores: F(2, 108) = 7.55, *p* =0.0008). Tukey post hoc: control vs. SD-LBP group (*p* = 0.0005); NS-LBP vs. controls (*p* = 0.066); NS-LBP vs. SD-LBP (*p* =0.148). ANOVA on PC2 scores: F(2,108) = 30.091, *p* <0.0001), Tukey post-hoc: control vs. NS-LBP (*p* =0.0001); control vs. SD-LBP group (*p* <0.0001); NS-LBP vs. SD-LBP (*p* =0.0004).

Nonlinear PCA (NLPCA) was conducted by solving for the Gifi loss function through optimal scaling and alternating least squares as implemented in the princal () function of the Gifi package in R (Mair et al., 2019). All variables were ordinally restricted limiting to monotonic transformations. Nonlinear transformations were performed by a b-spline of degree two and three knots placed into the quartiles of the data. To determine the number of significant principal components to retain for further analysis, 1,000 permutations of the resulting NLPCA solution were performed using a permutation test (permD method with 1,000 permutations) with the overall goal to reduce the number of components while maximizing the variance accounted for (Torres-Espín et al., 2021). Based on the results of the permutation test the first 7 PCs had significant signal above random noise (Supplementary Figure S1 shows the results of the permutation test). The final number of 3 PCs were chosen for construct validation established by applying the following criteria: Kaiser rule (Kaiser, 1960), Scree plot (Cattell, 1966), and factor specification based on top PC standardized loadings (correlations between the vector defined by a variable and the PC with an absolute correlation coefficient ≥0.5). The stability of the NLPCA solution was assessed by performing bootstrapping on the approximations of the NLPCA solution generated through linear PCA on nonlinear transformations of the data. Specifically, 300 balanced bootstrap samples were randomly drawn and 300 PCA solutions generated. Pattern matching statistics (root mean square difference in PC loading patterns, the coefficient of congruence, the Pearson product moment correlation coefficient) were calculated for the first 3 PCs between the PCA solution and each one of the bootstrapped PCA, generating an empirical distribution for each metric for each PC. The average and non-parametric 95% confident interval for each metric was calculated (Supplementary Table S1 shows the bootstrapping results). Following construct validation which is an expertise-guided process of determining whether the pattern of variable association determined by the loadings conform to an explainable interpretation based on the current knowledge and understanding of these variables, the first 2 PCs are discussed as containing pertinent information (Figure 2). The full list of loadings is provided in the barmap plot in Supplementary Figure S2.

ANOVA was used to determine the between-group difference of the stable principal component scores as well as patient demographics (age, BMI, VAS scores, and Oswestry Disability Index (ODI)). Pairwise comparisons were conducted by Tukey’s post hoc test. Chi-square statistic was used to asses between-group differences in participant sex distribution. Linear regression models (lm () function in R) were used to assess the relationship between principal components (PC1, PC2, and PC3) and patient-reported outcomes of pain (Visual Analogue Scale, VAS) and ODI with a covariate adjustment for age and body mass index (BMI). The absence of collinearity between the PCs, age, and BMI was confirmed with the variance inflation factor (vif () function in the car R package (Fox and Weisberg, 2019). Statistical significance for all analysis was set at α = 0.05.

## Results

### Participant Demographics

The analysis was performed on a convenience sample of patients with non-specific LBP, patients with adult spinal deformity, and healthy participants. There was no significant difference in male vs. female sex distribution across groups. There were significant between-group differences in age and BMI (control and NS-LBP groups only). Patients with adult spinal deformity had significantly higher VAS scores than patients with NS-LBP, however, there were no significant differences in ODI between the two patient groups. Neither VAS nor ODI measures were assessed in healthy controls, therefore, the control group was not included in the patient-reported outcome analysis (Table 1).

### NLPCA on the Biomechanical Variables

Unsupervised machine learning on full body biomechanics (Figures 1A,B) identified divergent movement strategies in LBP patients compared to healthy controls (Figures 2). The NLPCA workflow consists of multiple steps starting from raw participant-level data curation (Figure 1C), which then is subjected to optimal scaling transformation (Figure 1D), unsupervised pattern detection (Figures 1E,F), and expert-augmented construct validation (Figure 1G). This helps identify biomechanical parameters related to pain, enabling us to cluster participants based on their movement phenotypes (Figures 1H,I). Two global movement strategies represented by the principal components were deemed to capture a significant proportion of the variance (see methods for details).

### PC1: Reduced Full Body Kinetics, Hip, and Flexed Standing Posture Characterize Patient STS

The first principal component (PC1) accounted for 36.8% of variability within the dataset (Figure 2A). PC1 captured three different components of STS associated with either the control participant strategy (positive loadings) or patient movement (negative loadings). Maximum torso, pelvis, and thigh kinematics (accelerations and velocities) in the world and body frame, as well as peak L5S1, hip, and knee joint kinetic variables had significant positive correlations with PC1. World thigh maximum flexion angle, L5S1, hip minimum flexion angles, and knee minimum flexion torque had significant negative correlations with PC1 (Figure 2A). Based on this loading pattern, PC1 was regarded as a “vertical rise” STS strategy (positive loadings = full body vertical kinetics) and final standing posture (negative loadings = joint/segment kinematics).

There was a significant group difference in PC1 scores (F (2, 108) = 7.55, *p* =0.0008) (Figure 1B). Specifically, control participants had significantly greater PC1 scores as compared to SD-LBP group (*p* = 0.0005). There were no significant differences in PC1 scores between NS-LBP and controls (*p* = 0.066), and the two patient groups (*p* =0.148). Taken together these results suggest that lower full body kinetics and forward flexed standing posture are the main differentiating/defining features of SD-LBP patient STS movement when compared to healthy control participants.

### PC2: Leaning Stand-Up Strategy Differentiates Across the Two Patient Groups and Healthy Controls

The second principal component (PC2) accounted for the next 19.3% of variability in the data (Figure 1C). PC2 loadings featured several kinematic, kinetic, dynamic, and muscle force variables and for interpretability purposes, the loadings were grouped based on their biomechanical identity as following. Positive loadings included: world torso and pelvis maximum flexion angle, L5S1 and hip maximum flexion and maximum sagittal vertical axis (SVA) characterize body flexion or *forward-leaning;* thigh and shank maximum world anterior acceleration, shank maximum world anterior velocity, shank maximum body anterior and vertical velocities altogether encompassing *lower limb forward kinetics*; L5S1 lumbar anterior shear and compressive maximum forces, L5S1 sacral compressive maximum force and L5S1 extensor muscle maximum force which characterize *spine loading*; L5S1 and hip maximum flexion powers and L5S1 and hip maximum extension torques representing *total effort*; pelvis and torso maximum body posterior accelerations and velocities, thigh maximum world posterior velocities capturing *posterior body swing* (Figure 2C). The variety of biomechanical modules captured by PC2 comprises a leaning stand-up strategy and its biomechanical consequences (e.g., overall effort and spinal loading). There was a significant group difference in PC2 scores (F (2,108) = 30.091, *p* <0.0001) (Figure 2D). The control group had significantly lower PC2 scores as compared to NS-LBP (*p* =0.0001) and SD-LBP group (*p* <0.0001). NS-LBP has significantly lower PC2 scores as compared to SD-LBP (*p* =0.0004). The positive PC2 scores in the patient groups suggest that the positive PC2 loadings are more descriptive of the patient participants’ STS strategy. Thus, patients utilize a leaning forward–swing back STS strategy that is more laborious (requires greater extension torques) and more strenuous (results in higher spine loadings) (Figure 2E). Overall, NLPCA has separated the control and 2 patient groups into three overlapping but distinct clusters across PC1 and PC2 space (Figure 2F).

### PC3: A Complex, Defuse Construct

The third principal component (PC3) captured 10.4% of variability in the data. Kinematic, dynamic as well as kinetic variables have loaded into PC3 in a less cohesive fashion, capturing the remaining STS movement components. Positive loadings captured downward or caudal velocity of the pelvis and thigh body segments, the resulting flexion torque around the L5S1, knee and hip joints as well as world torso and pelvis maximum extension angles (leaning back). Negative loadings captured lower body maximum kinematics (acceleration and deceleration). Overall, these movement components do not link or group into a strategy as do PC1 and PC2 limiting the interpretability and establishment of its construct validity (Supplementary Figure S3A). Furthermore, there were no significant group differences in PC3 scores (F (2,108) = 1.78, *p* = 0.17) (Supplementary Figure S3B); therefore, they were excluded from further analysis.

### PC4–PC7 Constructs

The construct PC4–PC7 were excluded from the main analysis following Kaiser and Scree plot rules. However, based on the permutation test PC4–PC7 have significant loadings/information. P7 did not contain any loadings above 0.5 and was excluded from further investigation. The loadings of PC4–PC6, which together account for additional 16.1% variance in the data and capture the information related to participants ankle biomechanics, are reported in Supplementary Figure S4.

### Reduced Body Kinetics Predict Pain and Disability

**FIGURE 3:**
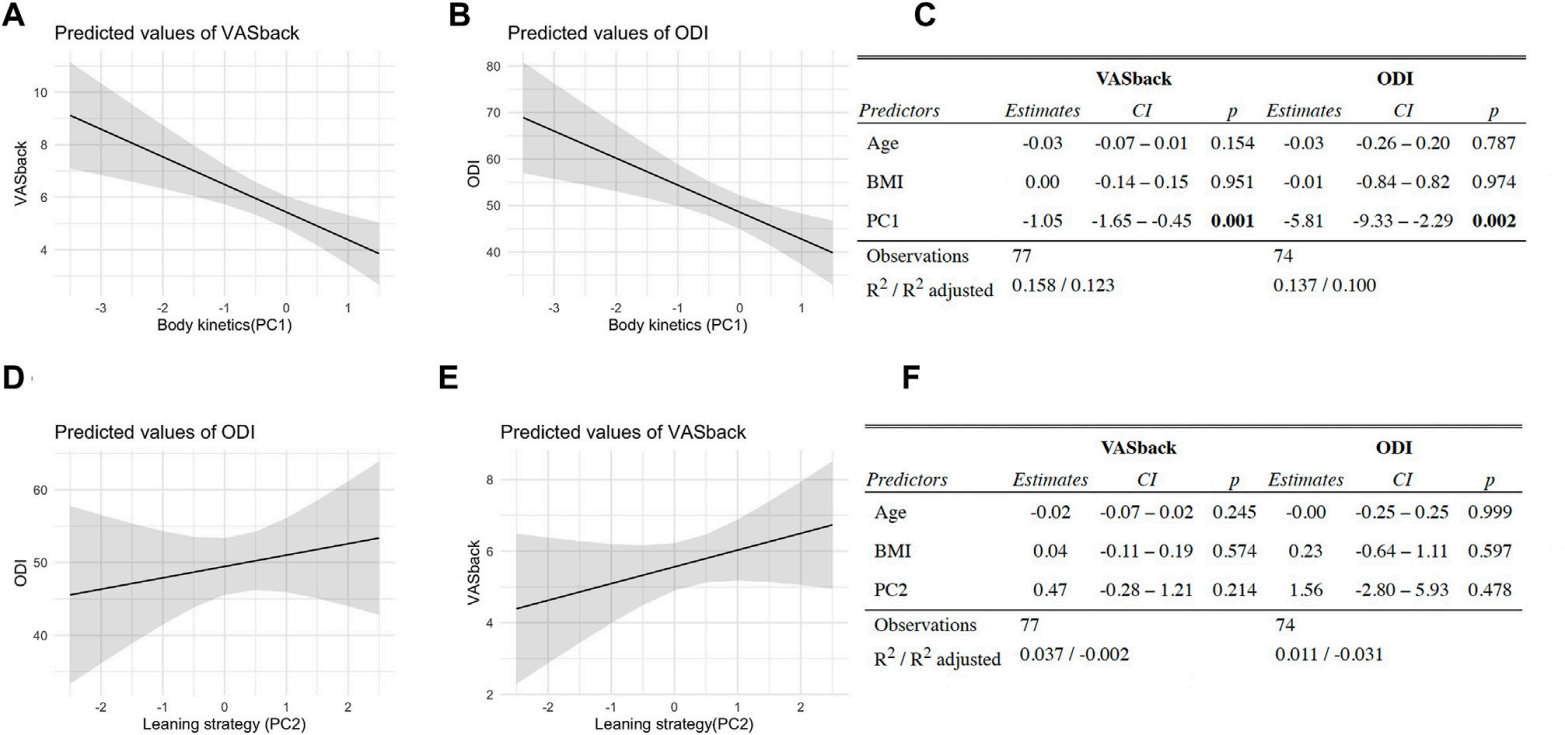
Reduced body kinetics predict pain and disability. **(A)** Body kinetics (PC1 scores) vs. patient pain scores (VASback, measured using Visual Analogue Scale) linear regression model plot. **(B)** Body kinetics (PC1 scores) vs. patient-reported disability (ODI, measured using Oswestry Disability Index) linear regression model plot. **(C)** PC1 vs. VASback and ODI prediction model results. **(D)** Leaning strategy (PC2 scores) vs. VASback linear regression model plot. **(E)** Leaning strategy (PC2 scores) vs. ODI linear regression model plot. f. PC2 vs. VASback and ODI prediction model results. BMI and age were included as co-factors to correct for their potential confounding effect in the relationship between body kinetics and patient-reported outcomes.

Linear regressions were used to determine whether biomechanical parameters and/or compensatory movement strategies captured across PC1 and PC2 can predict patient-reported outcomes including pain (VAS) and disability (ODI). We found a significant association between PC1 and VAS, as well as PC1 and ODI (Figures 3A–C) when controlling for BMI and age by including them as co-factors. The higher PC1 scores corresponding to greater speed of movement had a negative correlation with VAS and ODI scores, suggesting that patients with worse pain and greater disability move slower. In contrast, there were no significant associations between PC2 and VAS or PC2 and ODI (Figures 3D–F).

## Discussion

Based on the well-established interaction between nociceptive and sensorimotor circuitries (Schomburg et al., 2001; Amann et al., 2013; Mandadi et al., 2013; Sidhu et al., 2017; Massé-Alarie et al., 2019), the goal of this study was to identify patient-specific movement strategies by applying unsupervised machine learning on a set of full body biomechanics of STS movement collected from a convenience sample of patients with non-specific LBP, patients with adult spinal deformity and healthy participants. The overarching objective of our work is based on the idea that divergent motor control strategies identified using quantitative biomechanical measures may be used to, first, develop a discriminatory biomarker for patient subgrouping based on the profile of their sensorimotor dysfunction. A longer-term goal is to set the stage for developing a predictive biomechanical biomarker that captures maladaptive movement patterns that contribute to LBP recurrence.

NLPCA on a set of comprehensive full body biomechanics data acquired during STS using a marker-less system has identified significantly different movement strategies across healthy controls and the two patient groups. Control strategy is characterized by higher rostral kinetics which are coupled across torso, pelvis and thigh body segments resulting in faster transmission of momentum and a vertical rise (Figures 2A–C). In addition to reduced body kinetics, another main biomechanical characteristic that sets SD-LBP patients apart from the controls is a flexed or slouched final standing posture. Furthermore, both patient groups (NS-LBP and SD-LBP) initiate STS by leaning forward which is accompanied by increased lower limb forward kinetics, suggesting that patients move horizontally/forward before standing up (Figures 2B,C). Patients complete STS by swinging their body back which is reflected in higher torso and pelvis posterior kinetics. Overall, this strategy is less efficient as it requires generation of higher extension torques, and, in addition, results in higher spinal loads.

The redundancy in the neuromusculoskeletal system allows for variations in movement and muscle recruitment for the same task. These compensatory strategies can be due to a lack of *reserve* or a balancing of multiple *movement objectives* such as maximizing stability or minimizing pain (van der Kruk et al., 2021). While interplay between these effects is complex, stereotyped compensation strategies can be observed in different populations. An example of movement compensation stereotypes can be seen in the STS maneuver. A person can use a combination of momentum generation, torso leaning, and hip-knee extension to rise from a chair. Individuals with low back pain have been found to have higher peak torso flexion angles (Coghlin and McFadyen, 1994) and slower torso flexion and extension velocities (Shum et al., 2005a; Sánchez-Zuriaga et al., 2011). These differences in movement suggest a reduction in the use of the momentum generation strategy and a higher reliance on torso leaning. To execute STS using vertical rise strategy, efficient and coordinated recruitment of sufficiently strong hip/knee extensors is required. Pelvis/thigh body segments are the primary dynamic movers when the task is to displace the body from a seated to standing position, whereas the predominant role of trunk muscles is to stabilize the spine to prevent significant deviation in posture during the vertical rise (Rodrigues-de-Paula Goulart and Valls-Solé, 1999). We found that patients, on the other hand, rely on torso/pelvis flexion/leaning forward and torso/pelvis swing back (captured by increased posterior velocities) to accomplish STS task. Given that this strategy is also associated with increased spinal loading, it seems counterintuitive that patients with LBP would adopt a leaning STS strategy. This discrepancy reveals a deficiency in the ability to generate vertical momentum which may be a result of weak hip or knee extensor muscles previously found to be associated with LBP (Cai and Kong, 2015). From biomechanical standpoint, exaggerated trunk flexion during STS reduces knee extensor moments (Doorenbosch et al., 1994). Furthermore, a study on STS strategies in a healthy elderly population identified a significant negative correlation between isokinetic knee strength and the trunk flexion amplitude during STS (Dehail et al., 2007).

**FIGURE 4:**
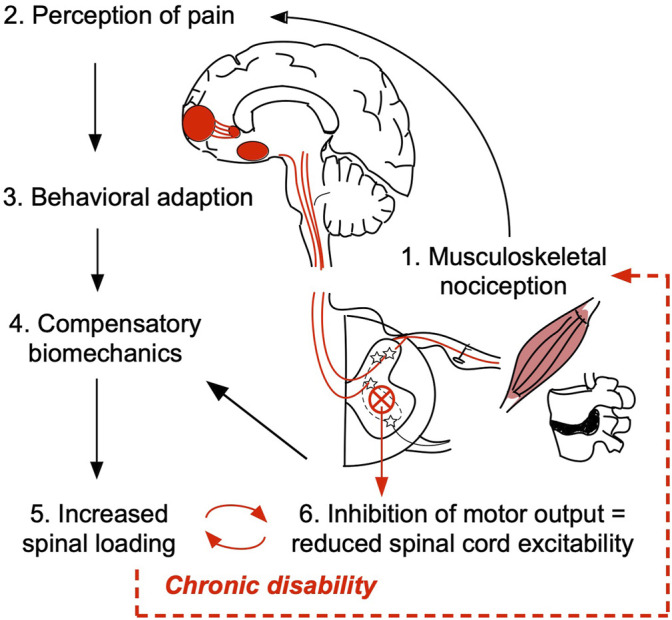
Working hypothesis of the relationship between pain/nociception and patient compensatory STS movement biomechanics. Damage in the peripheral tissue, such as muscle, ligament, or intervertebral disc may trigger the low back pain (LBP) onset (1). Upon initial injury, activation of primary nociceptive afferents triggers a cascade of action potentials across the central neural axis ultimately propagating the signal to the higher brain structures responsible for cognitive and emotional processing of pain (2). According to pain fear-avoidance model patients may consciously/volitionally seek to reduce physical activity and/or adopt compensatory strategies to avoid moving a painful joint (3 and 4). In this study, we determined that patients use a leaning forward–swing back sit-to-stand strategy that results in higher spinal loading (5). Presumably, the inefficient strategy/increased spinal loading maintains ongoing nociceptive tone. Persistent nociceptive signaling overtime results in decreased motor output and reduced spinal cord excitability (6) which at the subconscious level may contribute to the observed compensatory biomechanics, locking individuals with LBP in a viscous cycle of chronic disability.

Reduced body kinetics captured in PC1 associate significantly with VAS and ODI, suggesting that pain is the substrate for the reduction in movement speed and reduced physical function as the overall outcome in our study. The compensatory biomechanics may arise from a conscious decision to move slower and/or more cautiously consistent with pain fear-avoidance conditioning (Asmundson et al., 1999; Leeuw et al., 2007). In addition, assuming an ongoing nociceptive input from an aggravated spine joint/muscle, changes in biomechanics may result from a shift in the muscle recruitment patterns which occur at the spinal cord level (without volitional control) (Hodges and Richardson, 1999; Hodges and Tucker, 2011; Hodges and Smeets, 2015; Hodges et al., 2019). Furthermore, persistent nociceptive input results in a reduction of motor output and decreased spinal cord excitability (Gandevia, 2001; Le Pera et al., 2001; Nijs et al., 2012). It is likely that patients with LBP, thus, have a decreased capacity to recruit the extensor muscles necessary for STS which may be the reason for compensatory leaning strategy adaptation, slower movement speeds, as well as a flexed standing posture at the end of STS in LBP patients (overall reduced extensor tone) (Figure 4).

Surprisingly, PC2 scores did not correlate with either VAS or ODI, suggesting that NLPCA separated biomechanical parameters related to pain and physical dysfunction (PC1) from those that are not (PC2). Given the fact that PC2 captures part of the residual variance not explained by PC1, the separation based on movement biomechanics captured in PC2 is, therefore, mechanical in nature highlighting the reduced neuromuscular capacity revealed by demanding STS task (Papi et al., 2018). Both control (vertical rise) and patient (forward leaning) movement strategies have been described previously as different ways of getting up out of a chair (van der Kruk et al., 2021). However, there was no discussion of these strategies in the context of LBP in prior studies, or what implications these strategies might have on LBP development. Through computational modeling of full body biomechanics, we found that the leaning strategy results in increased spinal loading. Alternative to the hypothesis of nociceptive-afferent/pain-induced inhibition of motor output, is a possibility that the compensatory STS strategy (forwarding leaning) is utilized by the individuals with weak hip/lower body extensors (Kankaanpää et al., 1998; Nadler et al., 2002) before the onset of LBP. Overtime increased spinal loading may then lead to overuse low back injuries. Therefore, the weakness in hip/trunk stabilizing muscles and/or deficits in the neuromuscular control of pelvis/back might contribute to the risk of low back injury.

It remains to be empirically determined whether, first, the leaning strategy is related to hip/trunk muscle weakness and second, whether the leaning strategy can be used as a biomechanical biomarker or a predictor of future LBP onset in asymptomatic individuals. Although currently the transition from acute to chronic low back pain condition is poorly understood, acute injury is the first step towards a cascade of events that are multidimensional in nature and become difficult to disentangle. Given the high propensity of LBP and its persistence after the initial episode, target programs for LBP prevention and specific movement education may need to be implemented throughout an individual’s lifespan, for example, as part of a healthy lifestyle in the workplaces, similar to what has been done in regard to cardiovascular health and fitness.

### Limitations

There are several important limitations in this study. The analysis was performed on a convenience sample, in which age and BMI were not controlled. The two patient groups were significantly older and had higher BMI as compared to control subjects. Aging is associated with increased BMI, as well as overall reduced fitness, including muscle capacity. To control for the potential confounders, age and BMI were included as co-factors in the linear regressions of PC scores with VAS and ODI outcomes, and no significant contribution of either age or BMI was found on the significant correlation between PC1 scores with VAS and ODI, suggesting that pain and disability are more strongly related to the observed movement phenotypes than either age or BMI. In addition, LBP is a multidimensional heterogeneous disease and, therefore, patient phenotyping using even the most comprehensive biomechanics measures does not fully address the multifactorial nature of LBP. Deep patient phenotyping should be ideally performed on many outcome measures, including comprehensive patient-reported outcomes collected through validated NIH questionnaires (e.g., PROMIS), muscle electromyography, neuroimaging, quantitative sensory testing, etc., that capture the different aspects of the biopsychosocial model of LBP.

## Conclusions

Despite the limitations, this study identified a full body-based biomechanical biomarker derived from STS which is a highly relevant movement in the context of activities of daily living. Increased spinal loading, associated with the inefficient leaning STS strategy observed in patients with LBP, may underly the LBP chronicity. Overall, the leaning STS strategy appears counterintuitive to LBP condition, pointing to the underlying extensor muscle weakness of the primary movers (knee/hip extensors) during STS in patients with LBP. We suggest that LBP patient physical therapy-based rehabilitation efforts should include lower extremity strengthening with the goal to correct biomechanical insufficiency identified in patients with LBP.

## Data Availability

The raw data supporting the conclusions of this article will be made available by the authors, without undue reservation.
