# Supplementary material for: Unsupervised Machine Learning on Motion Capture Data Uncovers Movement Strategies in Low Back Pain
Source: Front Bioeng Biotechnol. 2022 Apr 14;10:868684. doi: 10.3389/fbioe.2022.868684 (PMC9047543; doi:10.3389/fbioe.2022.868684)
Supplement: Supplementary file 1 [file DataSheet2.PDF]

1 Libraries used in analysis
2 Dataset curation
3 NLPCA
4 Permutation
5 Bootstrapping
6 Figure 2
7 Supplemental Fig. 3b
8 % variance explained for PC1-PC6
9 Patient reported outcomes (PRO) + PC scores dataset curation
10 Univariate linear models
11 Table1 Demographics
12 PC4-PC7 - supplemental material
13 Supplemtanl Fig. 4 (PC4-PC6 bar plots) + ANOVA tests
14 Session info

Supplementary code for: Unsupervised machine learning on motion capture data uncovers movement strategies in low back pain

Anastasia V. Keller, Abel Torres-Espin, Thomas A. Peterson, Jacqueline Booker, Conor O'Neill, Jeffrey C Lotz, Jeannie F Bailey, Adam R. Ferguson, Robert P. Matthew

1 Libraries used in analysis

```
library(tidyverse)
library(syndRomics)
library(plotly)
library(scatterplot3d)
library(car, rgl)
library(Gifi)
library(psych)
library(ISLR)
library(splines)
library(sjPlot)
library(table1)
library(knitr)
library(tableHTML)

#install.packages("tableHTML")
```

2 Dataset curation

2.1 Loading datasets

```
Biomechanics <- read.csv("data/AllData.csv")
etiology <- read.csv("data/masterfile_clinical.csv")
```

2.2 Filtering outliers

```
biomechanics_nonorm <-Biomechanics%>%
  filter(Cohort%in% c("LBP", "Spine", "Controls"))%>%
  filter(!SID=="C051"|!Trial == "2"|!Rep == "3",!SID =="C052"|!Trial =="2")

biomechanics_noOUT <- biomechanics_nonorm%>%
  filter(outlier(biomechanics_nonorm[,10:17])<40)
```

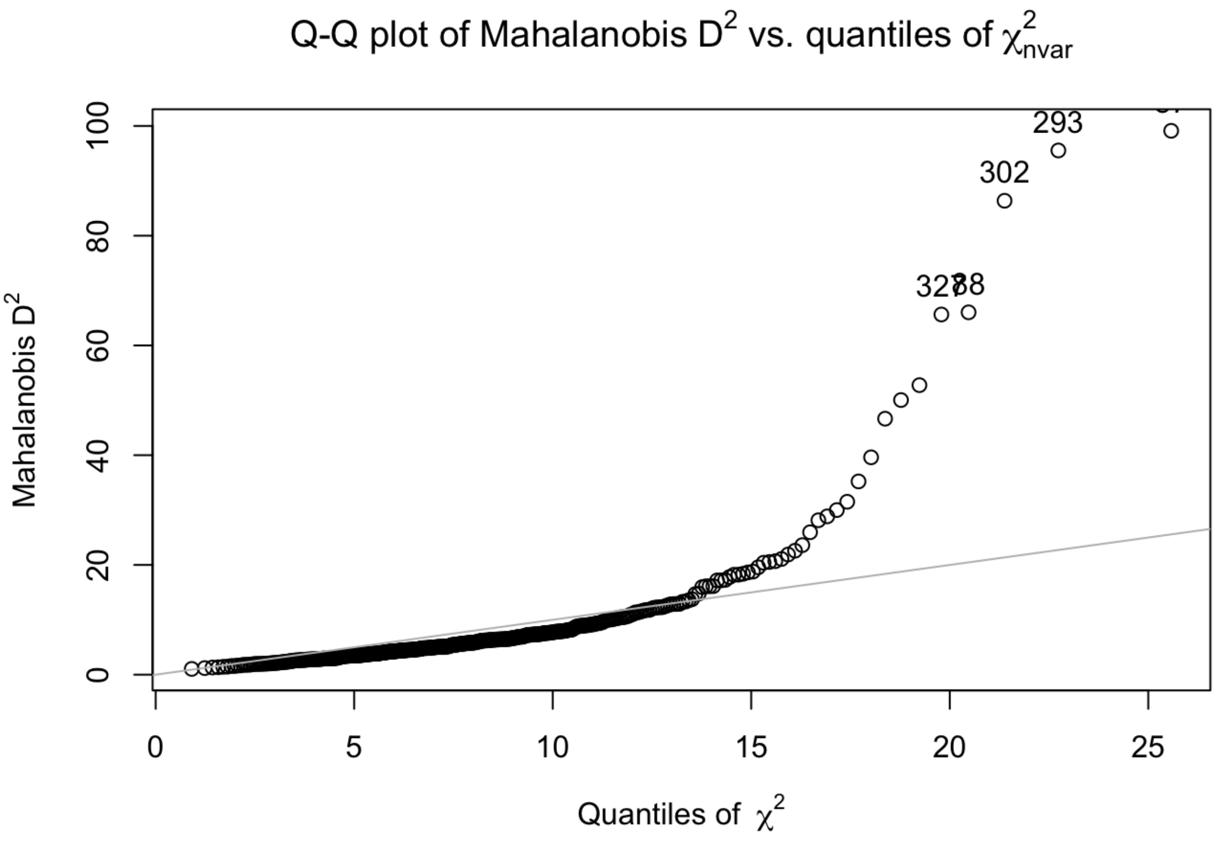

2.3 Filtering extra variables out

```
ave_trials_test <- biomechanics_noOUT[,str_detect(colnames(biomechanics_noOUT),
"NormHM|NormH|NormM|MaxSVANormH|SID|Age|Cohort|Rep|Trial|Sex|Height|Mass|AnkleM
axFlexAng|AnkleMinFlexAng|KneeMaxFlexAng|KneeMinFlexAng|HipMaxFlexAng|HipMinFle
xAng|L5S1MaxFlexAng|L5S1MinFlexAng|WorldShankMaxFlexAng|WorldShankMinFlexAng|Wo
rldThighMaxFlexAng|WorldThighMinFlexAng|WorldPelvisMaxFlexAng|WorldPelvisMinFle
xAng|WorldTorsoMaxFlexAng|WorldTorsoMinFlexAng|AnklePeakFlexDAng|AnklePeakExtDA
ng|AnklePeakFlexDDAng|AnklePeakExtDDAng|KneePeakFlexDAng|KneePeakExtDAng|KneePe
akFlexDDAng|KneePeakExtDDAng|HipPeakFlexDAng|HipPeakExtDAng|HipPeakFlexDDAng|Hi
pPeakExtDDAng|L5S1PeakFlexDAng|L5S1PeakExtDAng|L5S1PeakFlexDDAng|L5S1PeakExtDDA
ng")]

ave_trials_test <- ave_trials_test[,!str_detect(colnames(ave_trials_test),"T$|T
T$|TTT$")]
```

2.4 Participant matching between PROs and biomechanics datasets

```
demographics_test <- ave_trials_test%>%
  filter(!SID == "LBP0003", !SID =="LBP0027", !SID =="LBP0029", !SID =="BPC000
3", !SID =="C049", !SID =="C060", !SID == "C071", !SID =="C081",!SID == "C082",
!SID =="C083", !SID =="C084", !SID =="C085", !SID =="LBP0047")%>%
  select(Cohort, Age, Mass, Height, SID, Sex)%>%
  group_by(SID)%>%
  summarize_all(.funs = unique)
```

2.5 Averaging trials by SID

```
ave_trials_test <- ave_trials_test%>%
  select(-Cohort, -Rep, -Trial)%>%
  group_by(SID)%>%
  summarize_all(.funs = mean)

etiology_test <- etiology%>%
  select(Age, SID, Group)

biomechanics_test <- full_join(etiology_test, ave_trials_test, by ="SID")

biomechanics_test <- biomechanics_test%>%
  filter(!SID == "LBP0003", !SID =="LBP0027", !SID =="LBP0029", !SID =="BPC000
3", !SID =="C049", !SID =="C060", !SID == "C071", !SID =="C081",!SID == "C082",
!SID =="C083", !SID =="C084", !SID =="C085", !SID =="LBP0047")

demographics <-biomechanics_test%>%
  select(Group, Age.x, Age.y, Mass, Height, SID, Sex)#%>%
  #group_by(SID)%>%
  #summarize_all(.funs = unique)

groups <- rep("Control", nrow(demographics))
groups[demographics$Group == "Back Pain"] <- "NS-LBP"
groups[demographics$Group == "Surgery"] <-"SD-LBP"

ave_trials <- biomechanics_test%>%
  select (- Age.x, -SID, -Sex, -Age.y, -Height, -Mass, - Group)
```

Back to top

2.6 Variable sign change + name optimization

```
# determine which variables are below zero
##option 1

#in the future keep SID for all the data curation steps and use, for example av
e_trials[,-1] to remove SID from the data for preprocessing

filterby <- apply(X = ave_trials, MARGIN = 2, function(x){
  mean(x <0)
  })
##option 2
#ave_trials_min1 <- ave_trials%>%
# summarize_all(.funs = function(x) {
#    mean (x< 0)
#    })
# which variables at 95% consist of negative data points

ave_trials_ratio <- ave_trials[,filterby >0.5]

#ave_trials_ratio <- ave_trials_ratio%>%
  #select(-TorsoMaxBodyXAcc, -ShankMaxBodyXAcc)

# transforming negative variables into positive
ave_trials_transformed <-ave_trials
ave_trials_transformed[colnames(ave_trials_ratio)] <- ave_trials_transformed[co
lnames(ave_trials_ratio)]*(-1)
ave_trials_transformed$WorldTorsoMinFlexAng <-ave_trials_transformed$WorldTorso
MinFlexAng*(-1)



# renaming the variables
ave_trials_transformed<- ave_trials_transformed%>%
 dplyr::rename(L5S1MaxExtTorqueNormHM = L5S1MinFlexTorqueNormHM,
         L5S1MaxExtPowerNormHM = L5S1MinFlexPowerNormHM,
         L5S1SacralPosteriorShearMaxForceNormM =L5S1SacralAnteriorShearMaxForce
NormM,
         HipMaxExtTorqueNormHM = HipMinFlexTorqueNormHM,
         HipMaxExtPowerNormHM = HipMinFlexPowerNormHM,
         AnkleMaxExtPowerNormHM = AnkleMinFlexPowerNormHM,
         AnkleMaxExtTorqueNormHM = AnkleMinFlexTorqueNormHM,
         KneeMaxExtPowerNormHM = KneeMinFlexPowerNormHM,
         KneeMaxExtTorqueNormHm = KneeMinFlexTorqueNormHM,
         PelvisMaxBodyPosteriorDec = PelvisMinBodyXAccNormH,
         PelvisMaxWorldPosteriorDec = PelvisMinWorldXAccNormH,
         PelvisMaxBodyPosteriorVel = PelvisMinBodyXVelNormH,
         PelvisMaxBodyCaudalVel = PelvisMinBodyYVelNormH,
         PelvisMaxWorldDownVel = PelvisMinWorldYVelNormH,
         PelvisMaxWorldPosteriorVel = PelvisMinWorldXVelNormH,
         PelvisMaxBodyCaudalDec = PelvisMinBodyYAccNormH,
         PelvisMaxWorldCaudalDec = PelvisMinWorldYAccNormH,
         TorsoMaxBodyPosteriorVel = TorsoMinBodyXVelNormH,
         TorsoMaxBodyPosteriorDec = TorsoMinBodyXAccNormH,
         TorsoMaxWorldPosteriorDec = TorsoMinWorldXAccNormH,
```

```
          TorsoMaxWorldDownVel = TorsoMinWorldYVelNormH,
          TorsoMaxBodyCaudalVel = TorsoMinBodyYVelNormH,
          TorsoMaxBodyCaudalDec = TorsoMinBodyYAccNormH,
          TorsoMaxWorldCaudalDec = TorsoMinWorldYAccNormH,
          ShankMaxWorldCaudalDec = ShankMinWorldYAccNormH,
          ShankMaxBodyCaudalDec = ShankMinBodyYAccNormH,
          ShankMaxBodyPosteriorVel = ShankMinBodyXVelNormH,
          ShankMaxWorldPosteriorDec = ShankMinWorldXAccNormH,
          ShankMaxWorldPosteriorVel = ShankMinWorldXVelNormH,
          ShankMaxBodyPosteriorDec = ShankMinBodyXAccNormH,
          ShankMaxBodyCaudalVel = ShankMinBodyYVelNormH,
          ShankMaxWorldDownVel = ShankMinWorldYVelNormH,
          ThighMaxWorldCaudalDec = ThighMinWorldYAccNormH,
          ThighMaxBodyCaudalDec = ThighMinBodyYAccNormH,
          ThighMaxBodyPosteriorDec = ThighMinBodyXAccNormH,
          ThighMaxBodyCaudalVel = ThighMinBodyYVelNormH,
          ThighMaxWorldCaudalVel = ThighMinWorldYVelNormH,
          ThighMaxBodyPosteriorVel = ThighMinBodyXVelNormH,
          ThighMaxWorldPosteriorDec = ThighMinWorldXAccNormH,
          ThighMaxWorldPosteriorVel = ThighMinWorldXVelNormH,
          WorldPelvisMaxExtAng = WorldPelvisMinFlexAng,
          WorldThighMinExtAng = WorldThighMaxFlexAng,
          WorldThighMaxExtAng = WorldThighMinFlexAng,
          WorldTorsoMaxExtAng = WorldTorsoMinFlexAng)
```

Back to top

3 NLPCA

```
ave_trialsT_nlpca <- princals(as.matrix(ave_trials_transformed), ndim = 8, ordi
nal = TRUE, degrees = 2, knots = knotsGifi(as.matrix(ave_trials_transformed), t
ype ="Q"))


ave_trialsT_nlpca$objectscores[,1] <- ave_trialsT_nlpca$objectscores[,1]*(-1)
ave_trialsT_nlpca$loadings[,1] <- ave_trialsT_nlpca$loadings[,1]*(-1)

saveRDS(ave_trialsT_nlpca, "ave_trialsT_nlpca.Rds")
```

4 Permutation

```
ave_trialsT_nlpca <- readRDS("ave_trialsT_nlpca.Rds")

permut_pca <- prcomp(ave_trialsT_nlpca$transform)

ave_trialsT_permut_results <- permut_pc_test(permut_pca, ave_trialsT_nlpca$tran
sform, ndim = 20, P = 1000)
```

4.1 Supplemental Figure 1a (%VAR accounted for plot)

```
plot(ave_trialsT_permut_results, plot_resample = T, ndim = 1:10)
```

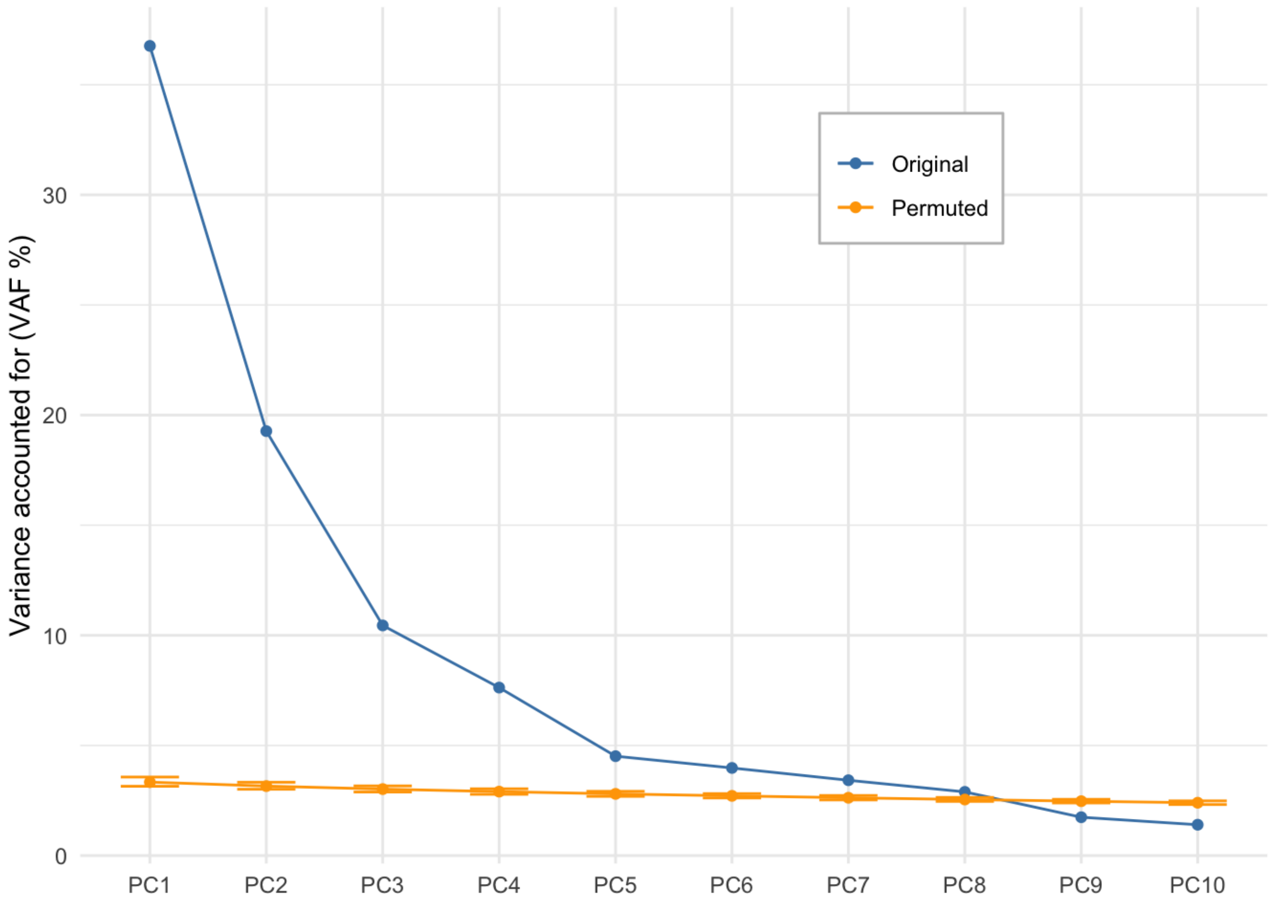

4.2 Supplemental Figure 1b (permutation stats resutls)

```
ave_trialsT_permut_results$results
```

```
##         original       mean     ci_low    ci_high      pvalue adj.p.value
## PC1  0.367630626 0.03334501 0.03146257 0.03565917 0.000999001 0.002497502
## PC2  0.192723104 0.03155179 0.03011977 0.03329088 0.000999001 0.002497502
## PC3  0.104492237 0.03018171 0.02886052 0.03162892 0.000999001 0.002497502
## PC4  0.076275875 0.02903759 0.02781564 0.03032692 0.000999001 0.002497502
## PC5  0.045131799 0.02801496 0.02688306 0.02917558 0.000999001 0.002497502
## PC6  0.039789257 0.02711933 0.02615283 0.02813600 0.000999001 0.002497502
## PC7  0.034205088 0.02625168 0.02526829 0.02728326 0.000999001 0.002497502
## PC8  0.028917632 0.02545563 0.02459822 0.02640380 0.000999001 0.002497502
## PC9  0.017413568 0.02469280 0.02388550 0.02554985 1.000000000 1.000000000
## PC10 0.014006044 0.02398693 0.02316839 0.02484410 1.000000000 1.000000000
## PC11 0.010232639 0.02326951 0.02253096 0.02403748 1.000000000 1.000000000
## PC12 0.007525907 0.02263438 0.02187542 0.02344575 1.000000000 1.000000000
## PC13 0.006836087 0.02200028 0.02125708 0.02273036 1.000000000 1.000000000
## PC14 0.006096139 0.02140475 0.02074656 0.02213637 1.000000000 1.000000000
## PC15 0.005307118 0.02081205 0.02010855 0.02149287 1.000000000 1.000000000
## PC16 0.003901246 0.02024440 0.01959070 0.02088370 1.000000000 1.000000000
## PC17 0.003486606 0.01970012 0.01905913 0.02033221 1.000000000 1.000000000
## PC18 0.003173053 0.01917089 0.01853313 0.01980532 1.000000000 1.000000000
## PC19 0.002810771 0.01865051 0.01806508 0.01929300 1.000000000 1.000000000
## PC20 0.002618006 0.01815683 0.01756738 0.01872675 1.000000000 1.000000000
```

Back to top

5 Bootstrapping

```
ave_trialsT_nlpca_BStrap_results <-pc_stability (permut_pca, as.data.frame(ave_
trialsT_nlpca$transform), B = 300, ndim = 3, s_cut_off = 0.1, test_similarity =
T, similarity_metric = 'all', sim = 'balanced')
```

5.1 Supplemental Table 1

```
Suppl_Table1 <-ave_trialsT_nlpca_BStrap_results$PC_similarity

Suppl_Table1
```

```
## $similarity_mean
## # A tibble: 3 x 5
##   PC    cc_index r_correlation   rmse s_index
##   <chr>    <dbl>         <dbl>  <dbl>   <dbl>
## 1 1        0.997         0.994 0.0458   0.987
## 2 2        0.996         0.995 0.0435   0.953
## 3 3        0.990         0.991 0.0457   0.933
##
## $similarity_ci_low
## # A tibble: 3 x 5
##   PC    cc_index r_correlation   rmse s_index
##   <chr>    <dbl>         <dbl>  <dbl>   <dbl>
## 1 1        0.992         0.983 0.0286   0.965
## 2 2        0.991         0.990 0.0281   0.918
## 3 3        0.966         0.969 0.0252   0.883
##
## $similarity_ci_high
## # A tibble: 3 x 5
##   PC    cc_index r_correlation   rmse s_index
##   <chr>    <dbl>         <dbl>  <dbl>   <dbl>
## 1 1        0.999         0.998 0.0815   1
## 2 2        0.998         0.998 0.0679   0.984
## 3 3        0.997         0.997 0.0865   0.969
```

5.2 Supplemental Figure 2

```
permut_results_suppl.fig <-plot(ave_trialsT_nlpca_BStrap_results, plot_resample
= T, ndim = 1:3, star_values =F)+
  theme_minimal(base_size = 8)

ggsave("Permut_results.pdf", permut_results_suppl.fig)
```

Back to top

6 Figure 2

SyndRomics plots in figure 2a and 2 c were created based on the loadings of each PC first visualized with the heatmap plots

6.1 PC1 loadings heatmaps

(results used to create plot in Fig 2a)

```
ave_trialsT_loadingsD1 <-stand_loadings(ave_trialsT_nlpca, ave_trials_transform
ed)%>%
  arrange(PC1)%>%
  #select(PC1,PC2,PC3)%>%
  filter(abs(PC1)>=0.5)

heatmap_loading (ave_trialsT_nlpca, ave_trials_transformed,ndim=1, vars = rowna
mes(ave_trialsT_loadingsD1))
```

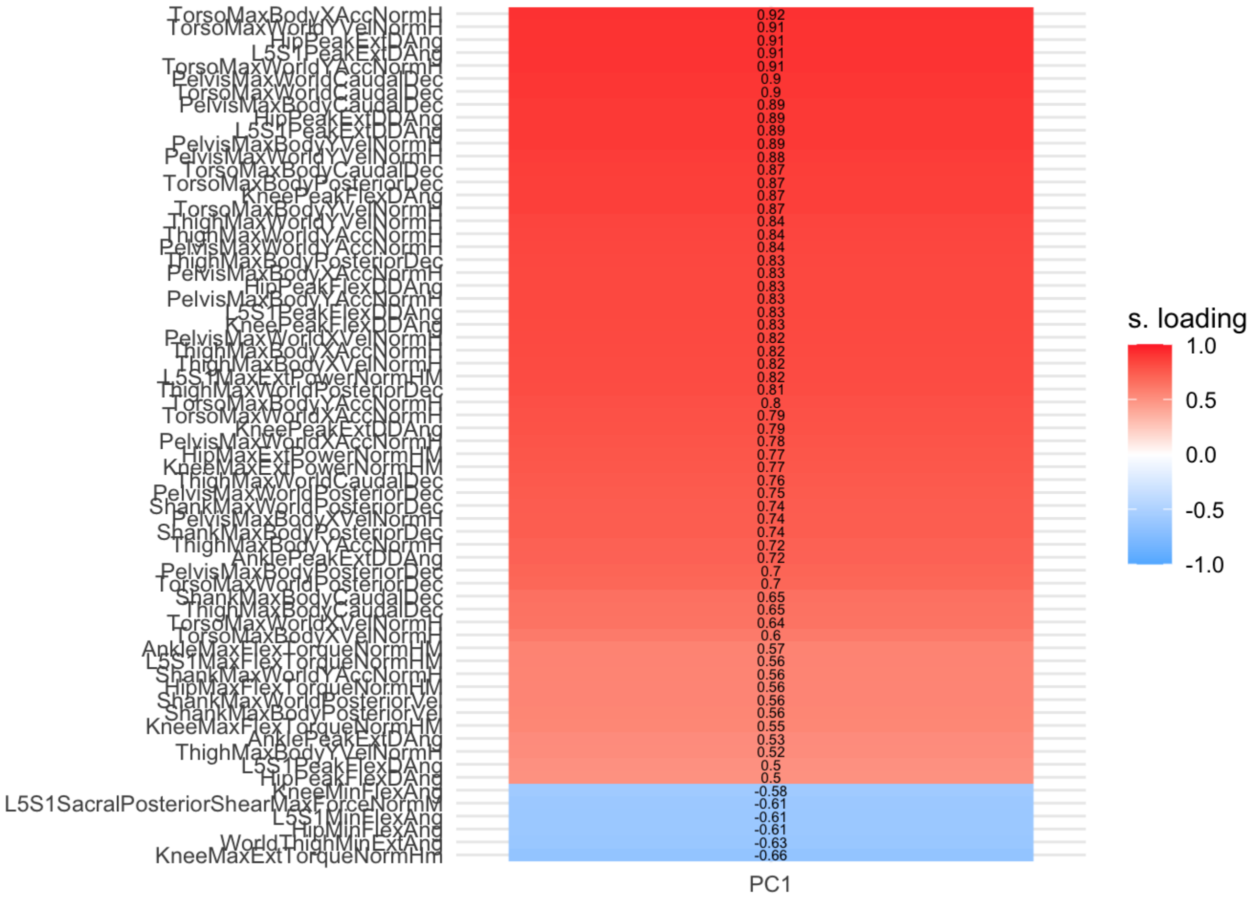

6.2 PC2 loadings heatmaps

(results used to create plot in Fig 2c)

```
ave_trialsT_loadingsD2 <-stand_loadings(ave_trialsT_nlpca, ave_trials_transform
ed)%>%
  arrange(PC2)%>%
  #select(PC1,PC2,PC3)%>%
  filter(abs(PC2)>=0.5)

heatmap_loading (ave_trialsT_nlpca, ave_trials_transformed,ndim=2, vars = rowna
mes(ave_trialsT_loadingsD2))
```

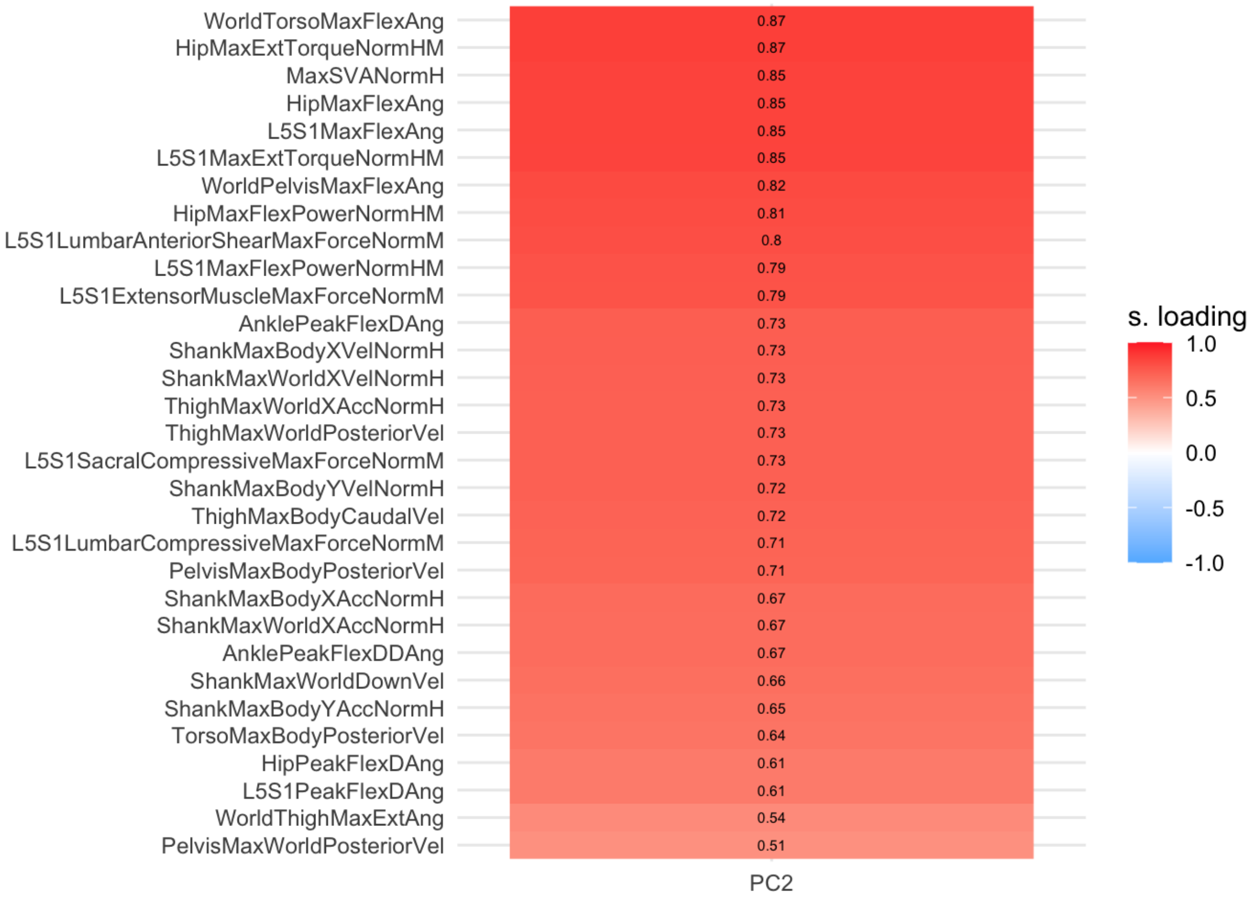

6.3 PC3 loadings heatmap

(results used to create plot in Supplemental Fig.3)

```
ave_trialsT_loadingsD3 <- stand_loadings(ave_trialsT_nlpca, ave_trials_transfor
med)%>%
  arrange(PC3)%>%
  filter(abs(PC3)>=0.5)

heatmap_loading (ave_trialsT_nlpca, ave_trials_transformed, ndim = 3, vars = ro
wnames(ave_trialsT_loadingsD3))
```

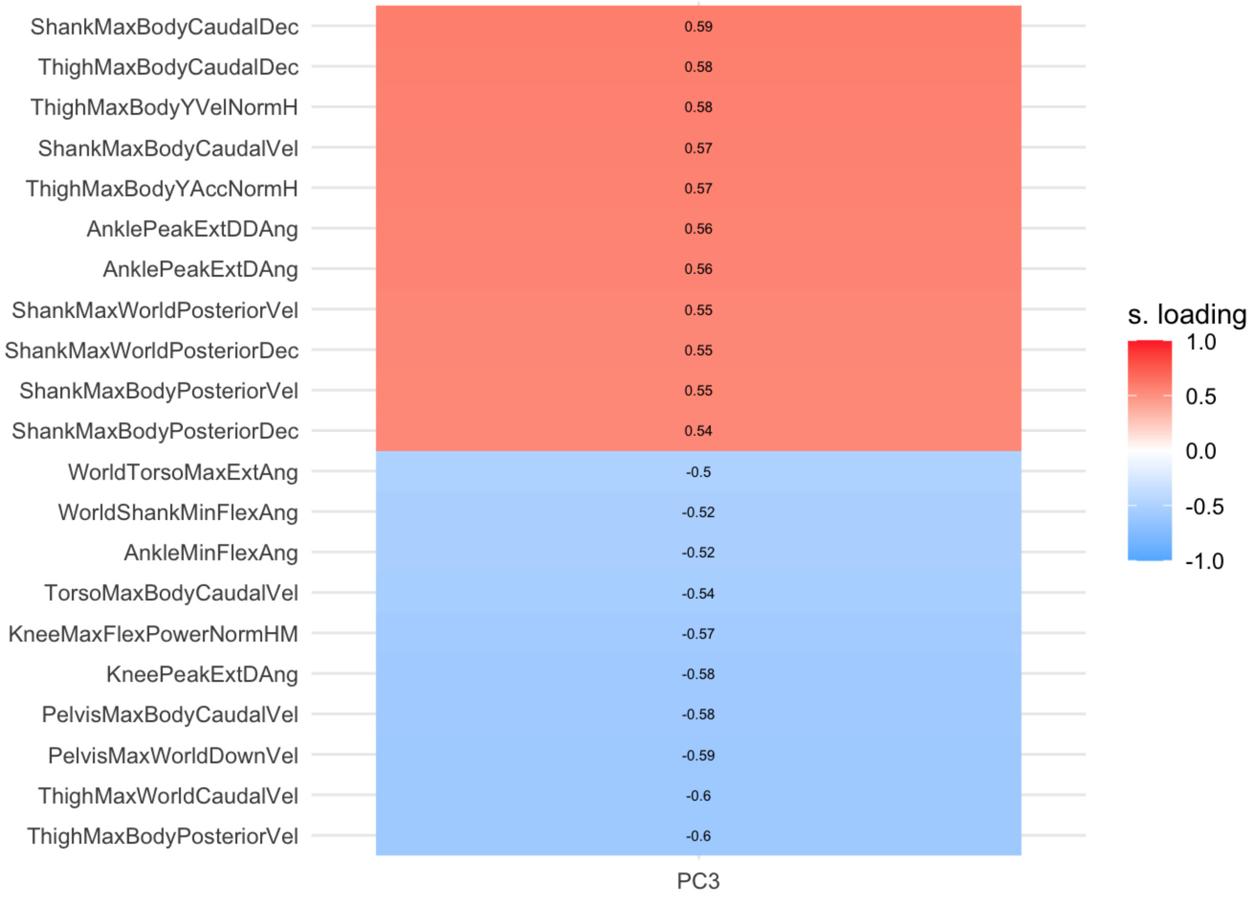

Back to top

6.4 Fig. 2f

(PC1 vs PC2 biplot)

```
ggplot(as.data.frame(ave_trialsT_nlpca$objectscores), aes(x = D1, y = D2, color
= groups, shape = groups))+
  geom_point(size=2.5)+
  theme_minimal(base_size = 20)+
  scale_color_manual(values = c("black", "blue3", "brown2"))+
  scale_shape_manual(values = c(16, 4, 15))+
  xlab("PC1")+
  ylab("PC2")+
  stat_ellipse(show.legend = FALSE, level=0.68, size = 0.8)+
  theme(legend.position = "bottom",legend.text = element_text(size = 14))+
  theme (legend.title = element_blank())
```

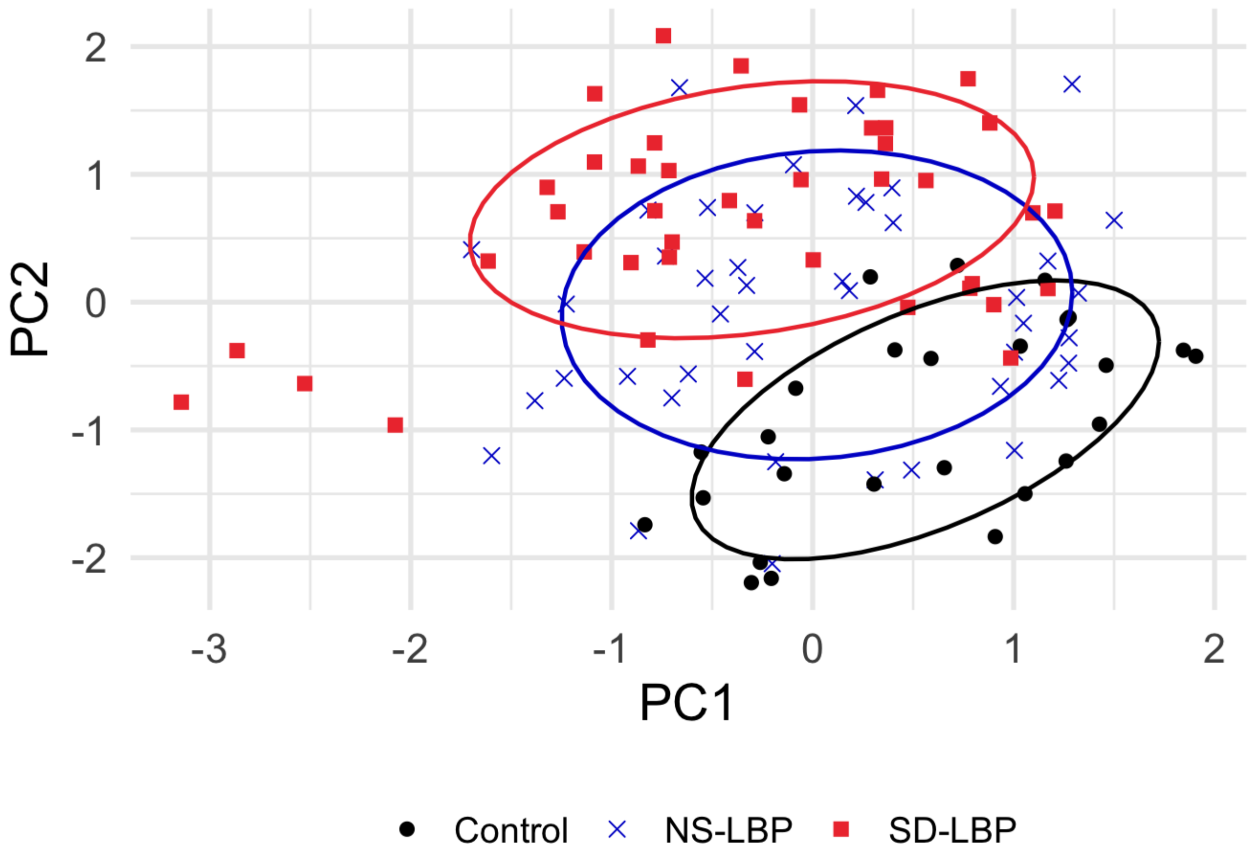


```
#labs(color=NULL)
```

6.5 Fig. 2b

(PC1 scores by group barplot)

```
ggplot(as.data.frame(ave_trialsT_nlpca$objectscores), aes(x=groups, y = D1, fil
l=groups))+
  geom_boxplot()+
  geom_point()+
  theme_minimal(base_size = 25)+
  xlab(NULL)+
  ylab("PC1")+
  #ylim(c(-15,15))+
  labs(fill=NULL)+
  scale_x_discrete(breaks=c(3,2,1))+
  theme(legend.position=c(.9,.75))+
  theme(legend.position = "left bottom",legend.text = element_text(size = 12))+
  theme (legend.title = element_blank())+
  scale_fill_manual(values = c("black", "blue3", "brown2"))
```

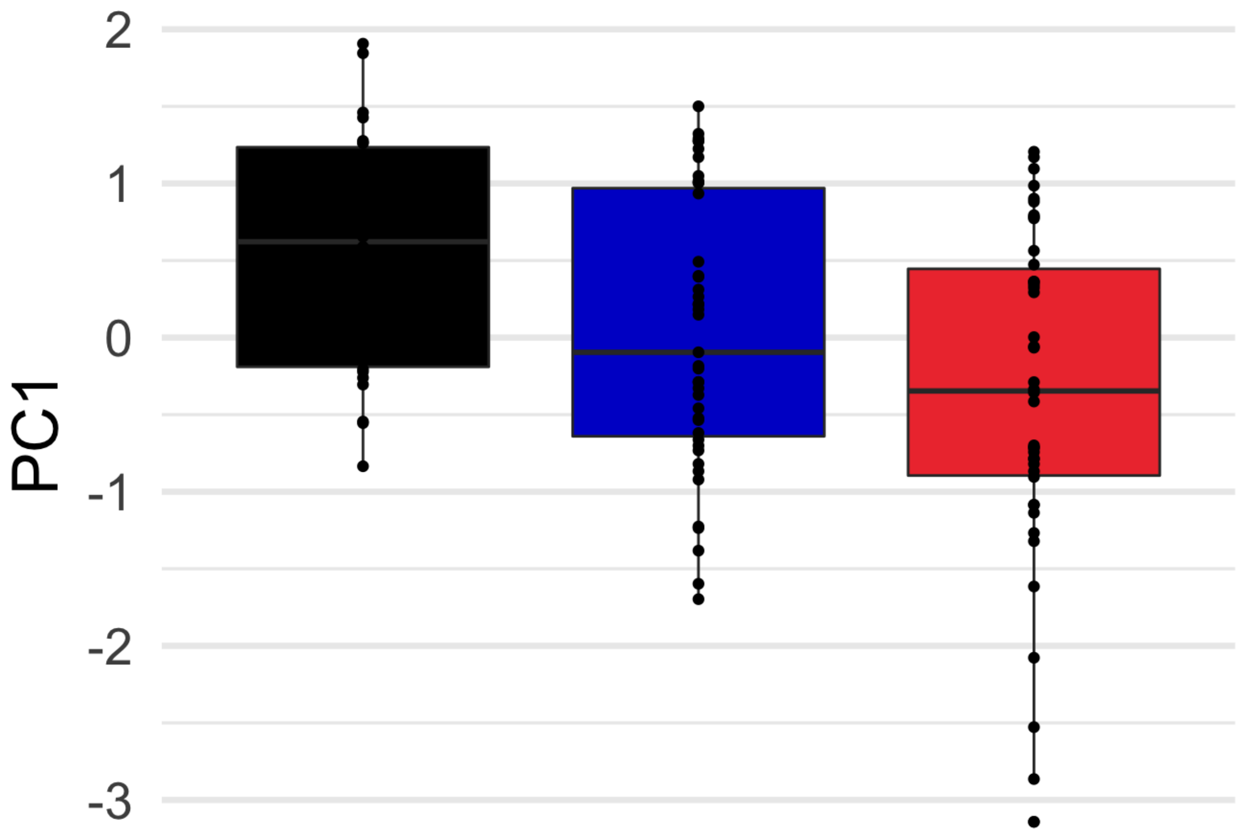

6.6 Fig. 2b stats

(ANOVA + posthoc on PC1 scores)

```
ANOVA_aveT_nlpca_PC1 <-aov(D1~groups, data = as.data.frame(ave_trialsT_nlpca$ob
jectscores))
anova(ANOVA_aveT_nlpca_PC1)
```

```
## Analysis of Variance Table
##
## Response: D1
##             Df Sum Sq Mean Sq F value    Pr(>F)
## groups       2 13.625  6.8125  7.5558 0.0008487 ***
## Residuals 108 97.375  0.9016
## ---
## Signif. codes:  0 '***' 0.001 '**' 0.01 '*' 0.05 '.' 0.1 ' ' 1
```

```
tukeyresults_aveT_PC1 <- TukeyHSD(x= ANOVA_aveT_nlpca_PC1)
TukeyHSD(x= ANOVA_aveT_nlpca_PC1)
```

```
##   Tukey multiple comparisons of means
##     95% family-wise confidence level
##
## Fit: aov(formula = D1 ~ groups, data = as.data.frame(ave_trialsT_nlpca$objec
tscores))
##
## $groups
##                       diff        lwr         upr     p adj
## NS-LBP-Control -0.5319626 -1.0925532  0.02862804 0.0667085
## SD-LBP-Control -0.9199379 -1.4830377 -0.35683820 0.0005201
## SD-LBP-NS-LBP  -0.3879754 -0.8775205  0.10156981 0.1484071
```

Back to top

6.7 Fig. 2d

(PC2 scores by group barplot)

```
ggplot(as.data.frame(ave_trialsT_nlpca$objectscores), aes(x=groups, y = D2, fil
l=groups))+
  geom_boxplot()+
  geom_point()+
  theme_minimal(base_size = 25)+
  xlab(NULL)+
  ylab("PC2")+
  #ylim(c(-15,15))+
  labs(fill=NULL)+
  scale_x_discrete(breaks=c(3,2,1))+
  theme(legend.position = "left bottom")+
  scale_fill_manual(values = c("black", "blue3", "brown2"))
```

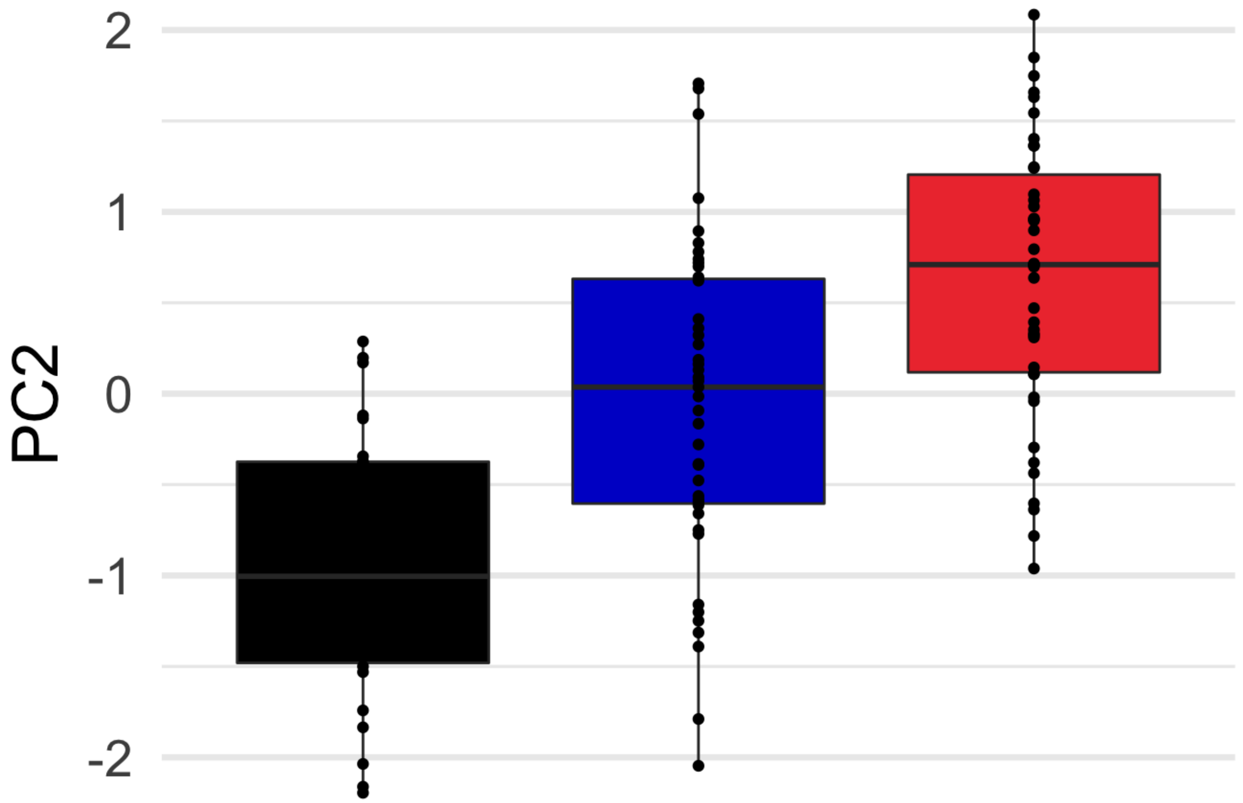

Back to top

6.8 Fig. 2d stats

(ANOVA + posthoc on PC2 scores)

```
ANOVA_aveT_nlpca_PC2 <-aov(D2~groups, data = as.data.frame(ave_trialsT_nlpca$ob
jectscores))
anova(ANOVA_aveT_nlpca_PC2)
```

```
## Analysis of Variance Table
##
## Response: D2
##            Df Sum Sq Mean Sq F value    Pr(>F)
## groups      2  39.72   19.86  30.091 4.099e-11 ***
## Residuals 108  71.28    0.66
## ---
## Signif. codes:  0 '***' 0.001 '**' 0.01 '*' 0.05 '.' 0.1 ' ' 1
```

```
tukeyresults_aveT_PC2 <- TukeyHSD(x= ANOVA_aveT_nlpca_PC2)
TukeyHSD(x= ANOVA_aveT_nlpca_PC2)
```

```
##   Tukey multiple comparisons of means
##     95% family-wise confidence level
##
## Fit: aov(formula = D2 ~ groups, data = as.data.frame(ave_trialsT_nlpca$objec
tscores))
##
## $groups
##                      diff       lwr      upr     p adj
## NS-LBP-Control 0.8726425 0.3930142 1.352271 0.0001011
## SD-LBP-Control 1.5679215 1.0861464 2.049697 0.0000000
## SD-LBP-NS-LBP  0.6952790 0.2764354 1.114122 0.0004154
```

7 Supplemental Fig. 3b

(PC3 scores by group barplot)

```
ggplot(as.data.frame(ave_trialsT_nlpca$objectscores), aes(x=groups, y = D3, fil
l=groups))+
  geom_boxplot()+
  geom_point()+
  theme_minimal(base_size = 25)+
  xlab(NULL)+
  ylab("PC3")+
  #ylim(c(-15,15))+
  labs(fill=NULL)+
  scale_x_discrete(breaks=c(3,2,1))+
  theme(legend.position = "left bottom")+
  scale_fill_manual(values = c("black", "blue3", "brown2"))
```

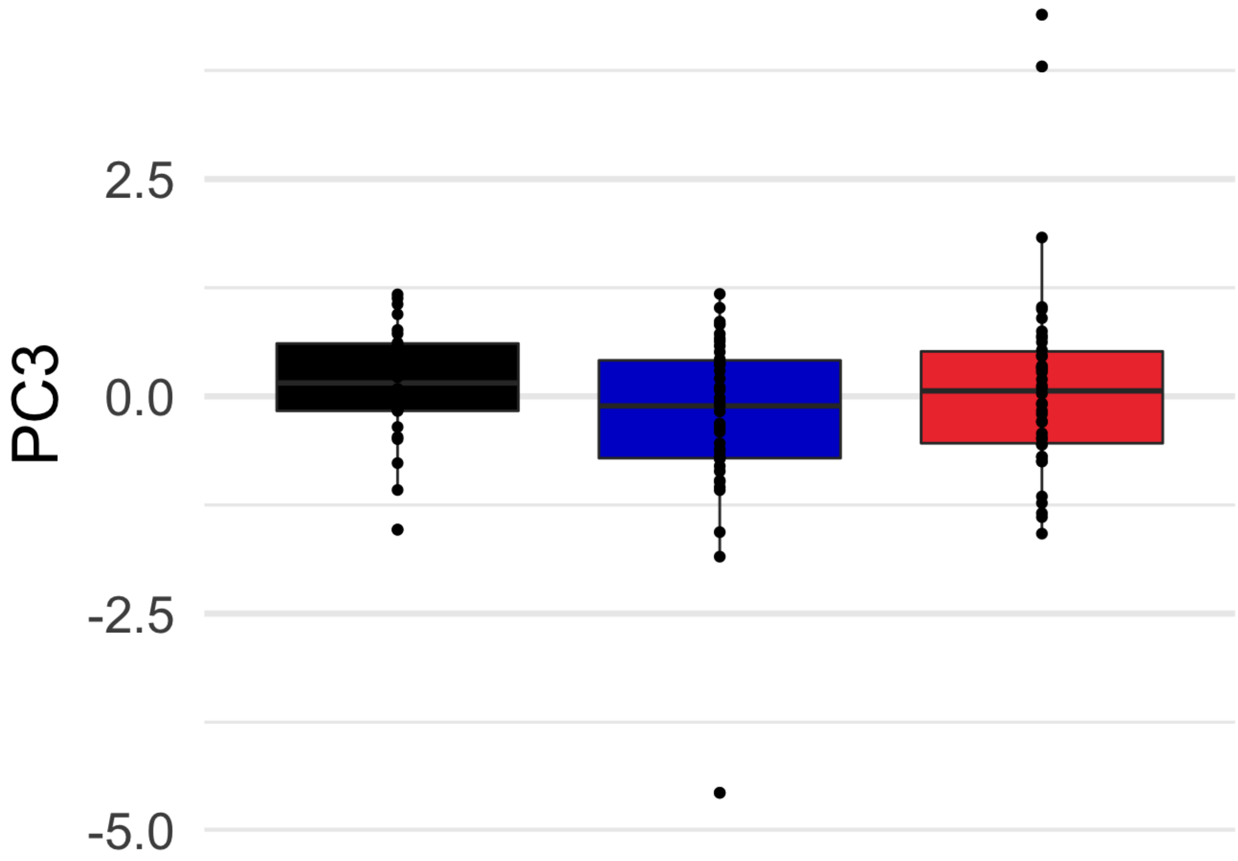

Back to top

7.1 Supplemetnal Fig. 3b

stats (ANOVA on PC3 scores)

```
ANOVA_aveT_nlpca_PC3 <-aov(D3~groups, data = as.data.frame(ave_trialsT_nlpca$ob
jectscores))
anova(ANOVA_aveT_nlpca_PC3)
```

```
## Analysis of Variance Table
##
## Response: D3
##            Df  Sum Sq Mean Sq F value Pr(>F)
## groups      2   3.564 1.78175  1.7911 0.1717
## Residuals 108 107.436 0.99478
```

8 % variance explained for PC1-PC6

as reported in the results, Figure 2, Supplemetnal Figures 3 and 4

```
aveT_nlpca_PCs <- syndromic_plot(ave_trialsT_nlpca, ave_trials_transformed, ndim = 6, cutoff = c(0.8, 0.5, 0.5, 0.5, 0.5, 0.5), text_size = 4)
aveT_nlpca_PCs_PC1 <- aveT_nlpca_PCs$PC1
aveT_nlpca_PCs_PC2 <- aveT_nlpca_PCs$PC2
aveT_nlpca_PCs_PC3 <-aveT_nlpca_PCs$PC3
aveT_nlpca_PCs_PC4 <-aveT_nlpca_PCs$PC4
aveT_nlpca_PCs_PC5 <-aveT_nlpca_PCs$PC5
aveT_nlpca_PCs_PC6 <-aveT_nlpca_PCs$PC6
```

9 Patient reported outcomes (PRO) + PC scores dataset curation

9.1 PC scores extraction

```
NSLBP_PCscores <- ave_trialsT_nlpca$objectscores
NSLBP_PCscores.SID <- cbind(demographics, NSLBP_PCscores)
```

9.2 PROs dataset curation

```
etiology <- read.csv("data/masterfile_clinical.csv")

etiology_select <- etiology %>%
  select(Group, SID, BMI, ODI2raw, VASback, VASRleg, VASLleg, VASneck, discogenic, myofascial, structuralscoliosis, degenerative.scoliosis, arthropathy, nervecompression)
```

Back to top

9.3 PROs + PC scores merge

```
clinicalvars <-full_join (etiology_select, NSLBP_PCscores.SID, by = "SID")

clinicalvars <-clinicalvars%>%
  #filter(Group %in% c("Back Pain"))%>%
  #na.exclude()%>%
 # select(-Group, -SID, -Cohort, -degenerative.scoliosis)
         #-D4, -D5, -D6, -D7, -D8)
  filter(!SID =="LBP0003",!SID == "LBP0027", !SID == "LBP0029", !SID == "BPC0003",!SID == "C049", !SID == "C060", !SID == "C071")%>%
  rename(ODI = ODI2raw)
```

10 Univariate linear models

10.1 Figure 3a

(VASback_PC1 plot)

```
VASback_age_bmi_D1 <- lm(VASback ~ Age.y +BMI + D1, data = clinicalvars)
summary(VASback_age_bmi_D1)
```

```
##
## Call:
## lm(formula = VASback ~ Age.y + BMI + D1, data = clinicalvars)
##
## Residuals:
##     Min      1Q  Median      3Q     Max
## -4.7177 -1.6140 -0.2078  1.8054  6.0651
##
## Coefficients:
##             Estimate Std. Error t value Pr(>|t|)
## (Intercept)  6.92326    2.32414   2.979 0.003926 **
## Age.y       -0.02816    0.01956  -1.439 0.154312
## BMI          0.00437    0.07158   0.061 0.951487
## D1          -1.05336    0.30137  -3.495 0.000809 ***
## ---
## Signif. codes:  0 '***' 0.001 '**' 0.01 '*' 0.05 '.' 0.1 ' ' 1
##
## Residual standard error: 2.656 on 73 degrees of freedom
##   (34 observations deleted due to missingness)
## Multiple R-squared:  0.1577, Adjusted R-squared:  0.1231
## F-statistic: 4.556 on 3 and 73 DF,  p-value: 0.005556
```

```
VASback_age_bmi_D1_plot <- plot_model(VASback_age_bmi_D1, type = "pre", terms =
"D1", show.data = F)

VASback_age_bmi_D1_plot +
  theme_minimal(base_size = 14)+
  xlab("Body kinetics(PC1)")
```

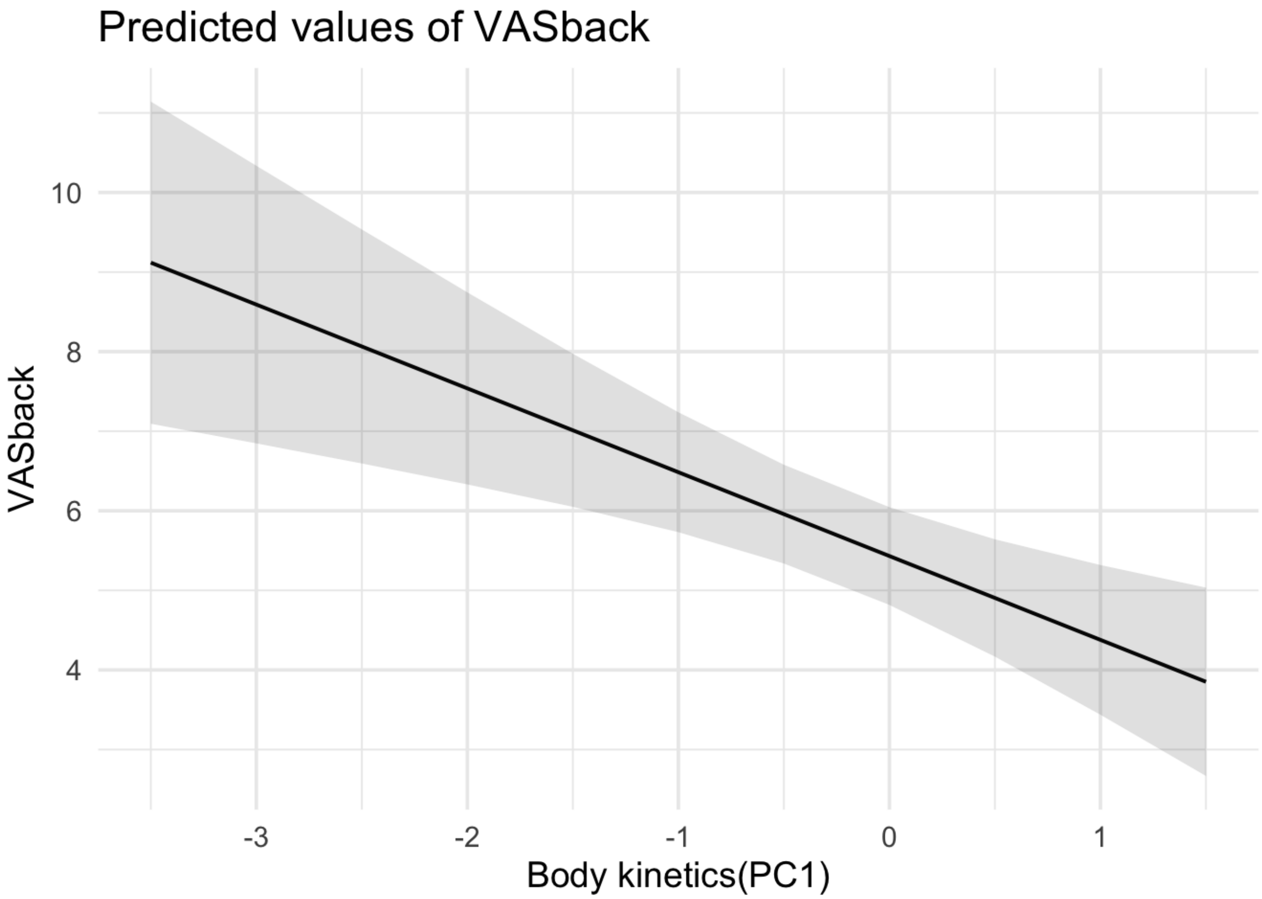

Back to top

10.2 Figure 3b

(ODI_PC1 plot)

```
ODI_age_bmi_D1 <- lm(ODI ~ Age.y + BMI + D1, data = clinicalvars)
summary(ODI_age_bmi_D1, correlation = T)
```

```
##
## Call:
## lm(formula = ODI ~ Age.y + BMI + D1, data = clinicalvars)
##
## Residuals:
##     Min      1Q  Median      3Q     Max
## -36.792 -10.060  -1.992   9.239  34.339
##
## Coefficients:
##             Estimate Std. Error t value Pr(>|t|)
## (Intercept) 50.69851   13.69089   3.703 0.000421 ***
## Age.y       -0.03090    0.11420  -0.271 0.787487
## BMI         -0.01364    0.41609  -0.033 0.973949
## D1          -5.80897    1.76477  -3.292 0.001563 **
## ---
## Signif. codes:  0 '***' 0.001 '**' 0.01 '*' 0.05 '.' 0.1 ' ' 1
##
## Residual standard error: 15.32 on 70 degrees of freedom
##   (37 observations deleted due to missingness)
## Multiple R-squared:  0.1374, Adjusted R-squared:  0.1004
## F-statistic: 3.716 on 3 and 70 DF,  p-value: 0.01533
##
## Correlation of Coefficients:
##       (Intercept) Age.y BMI
## Age.y -0.61
## BMI   -0.87        0.17
## D1    -0.18        0.13  0.18
```

```
ODI_age_bmi_D1_plot <- plot_model(ODI_age_bmi_D1, type = "pre", terms = "D1", s
how.data = F)
ODI_age_bmi_D1_plot +
  theme_minimal(base_size = 14)+
  xlab("Body kinetics (PC1)")
```

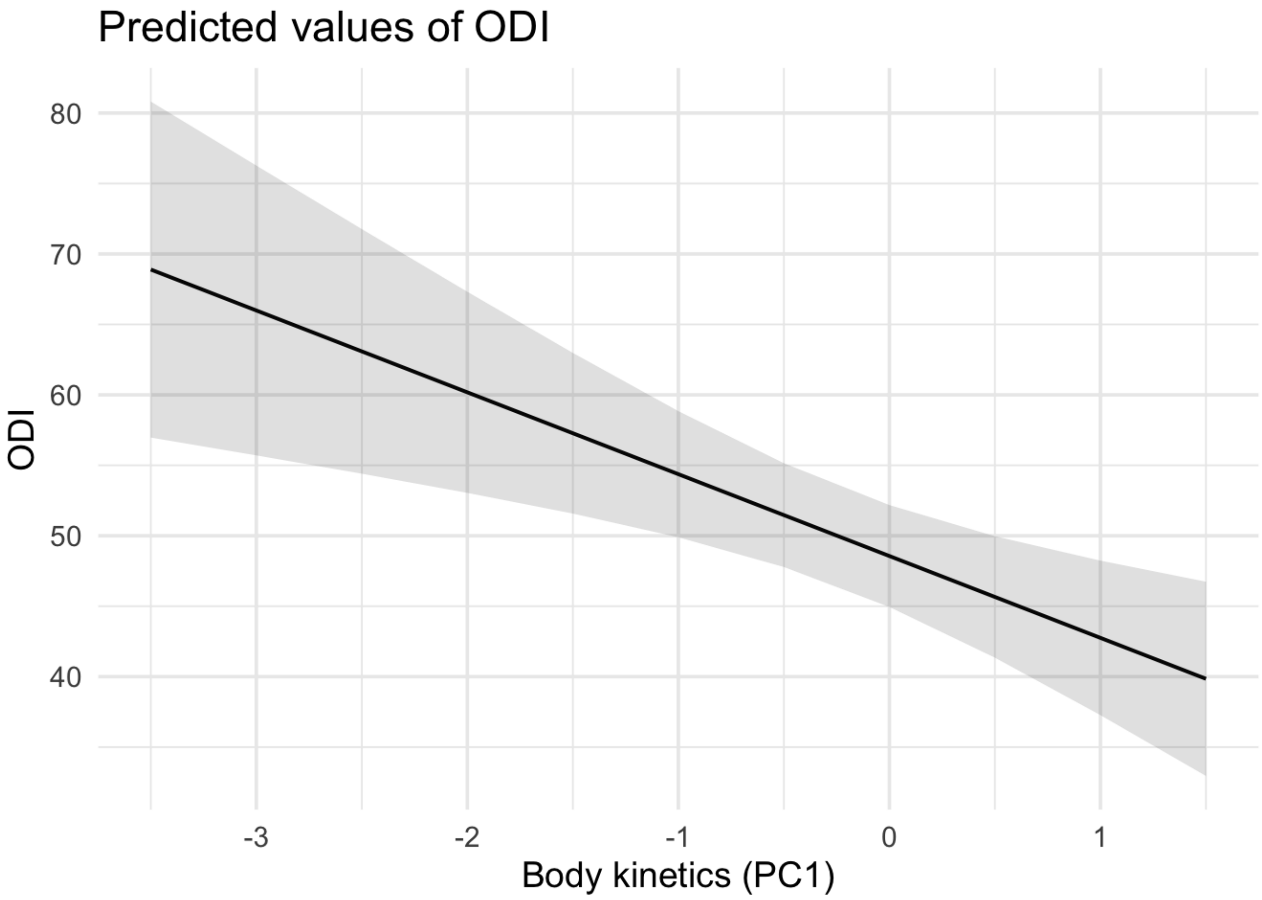

10.3 Figure 3c

(Linear model results)

```
tab_model(VASback_age_bmi_D1, ODI_age_bmi_D1, show.intercept = F, file = "VAS_O
DI_D1_table.html")
```

	VASback			ODI		
Predictors	*Estimates*	*CI*	*p*	*Estimates*	*CI*	*p*
Age.y	-0.03	-0.07 – 0.01	0.154	-0.03	-0.26 – 0.20	0.787
BMI	0.00	-0.14 – 0.15	0.951	-0.01	-0.84 – 0.82	0.974
D1	-1.05	-1.65 – -0.45	**0.001**	-5.81	-9.33 – -2.29	**0.002**
Observations	77			74		
R^2 / R^2 adjusted	0.158 / 0.123			0.137 / 0.100		

10.4 Fig 3d

(VASback_PC2 plot)

```
VASback_age_bmi_D2 <- lm(VASback ~ Age.y + BMI + D2, data = clinicalvars)
summary(VASback_age_bmi_D2)
```

```
##
## Call:
## lm(formula = VASback ~ Age.y + BMI + D2, data = clinicalvars)
##
## Residuals:
##    Min     1Q Median     3Q    Max
## -5.714 -2.176  0.119  1.909  5.111
##
## Coefficients:
##             Estimate Std. Error t value Pr(>|t|)
## (Intercept)  5.82174    2.46009   2.366   0.0206 *
## Age.y       -0.02478    0.02114  -1.172   0.2449
## BMI          0.04285    0.07596   0.564   0.5744
## D2           0.46799    0.37310   1.254   0.2137
## ---
## Signif. codes:  0 '***' 0.001 '**' 0.01 '*' 0.05 '.' 0.1 ' ' 1
##
## Residual standard error: 2.84 on 73 degrees of freedom
##   (34 observations deleted due to missingness)
## Multiple R-squared:  0.03748,    Adjusted R-squared:  -0.002078
## F-statistic: 0.9475 on 3 and 73 DF,  p-value: 0.4223
```

```
VASback_age_bmi_D2_plot <- plot_model(VASback_age_bmi_D2, type = "pre", terms =
"D2", show.data = F)

VASback_age_bmi_D2_plot +
  theme_minimal(base_size = 14)+
  xlab("Leaning strategy(PC2)")
```

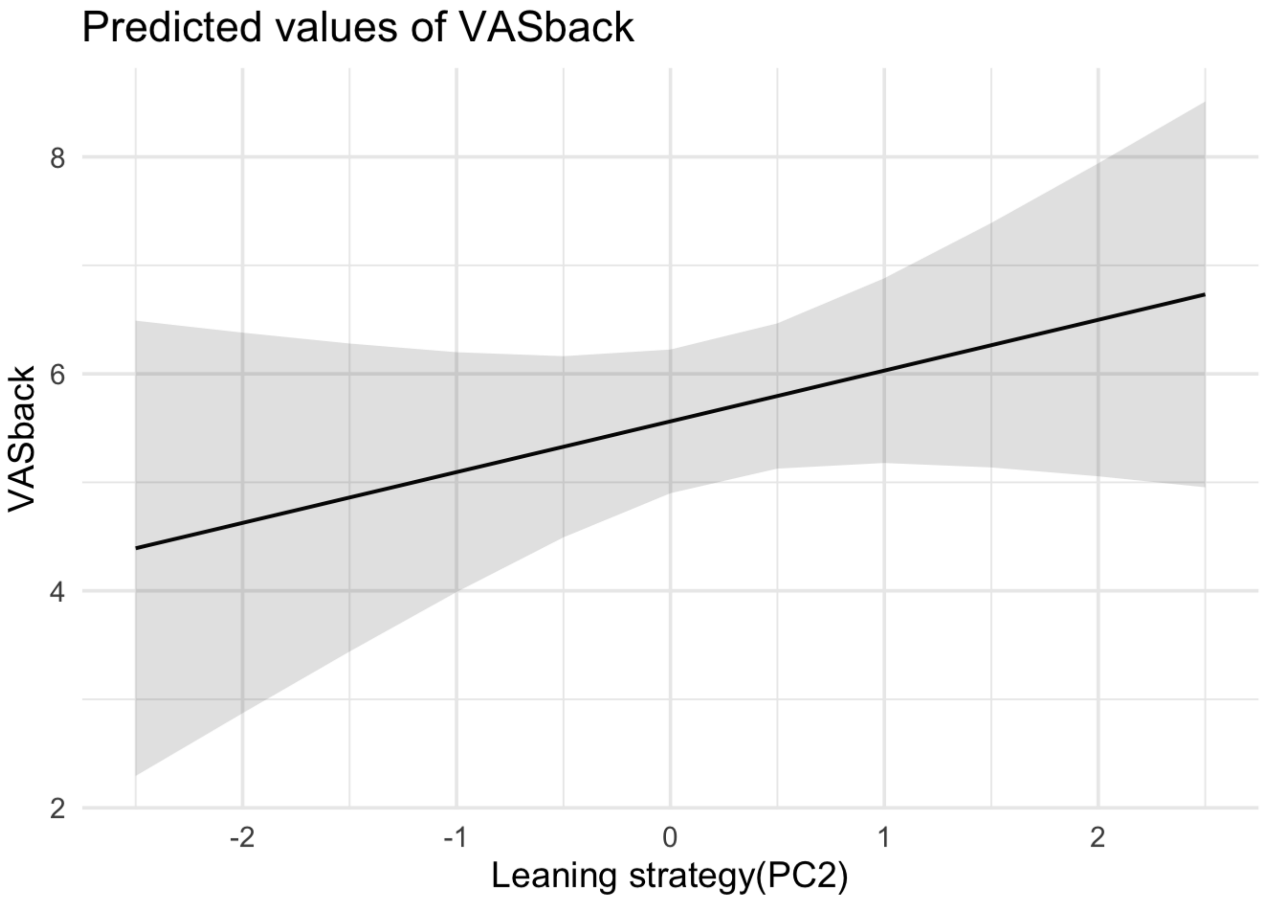

Back to top

10.5 Fig. 3e

(ODI_PC2 plot)

```
ODI_age_bmi_D2 <- lm(ODI ~ Age.y +BMI + D2, data = clinicalvars)
summary(ODI_age_bmi_D2, correlation = T)
```

```
##
## Call:
## lm(formula = ODI ~ Age.y + BMI + D2, data = clinicalvars)
##
## Residuals:
##     Min      1Q  Median      3Q     Max
## -42.791 -11.262   1.033  10.235  37.514
##
## Coefficients:
##               Estimate Std. Error t value Pr(>|t|)
## (Intercept) 43.1797999 14.4433717   2.990  0.00385 **
## Age.y       -0.0001564  0.1239037  -0.001  0.99900
## BMI          0.2326828  0.4386198   0.530  0.59745
## D2           1.5617652  2.1894363   0.713  0.47802
## ---
## Signif. codes:  0 '***' 0.001 '**' 0.01 '*' 0.05 '.' 0.1 ' ' 1
##
## Residual standard error: 16.4 on 70 degrees of freedom
##   (37 observations deleted due to missingness)
## Multiple R-squared:  0.01106,    Adjusted R-squared:  -0.03132
## F-statistic: 0.261 on 3 and 70 DF,  p-value: 0.8532
##
## Correlation of Coefficients:
##       (Intercept) Age.y BMI
## Age.y -0.60
## BMI   -0.87        0.14
## D2     0.06       -0.21  0.02
```

```
ODI_age_bmi_D2_plot <- plot_model(ODI_age_bmi_D2, type = "pre", terms = "D2", s
how.data = F)
ODI_age_bmi_D2_plot +
  theme_minimal(base_size = 14)+
  xlab("Leaning strategy (PC2)")
```

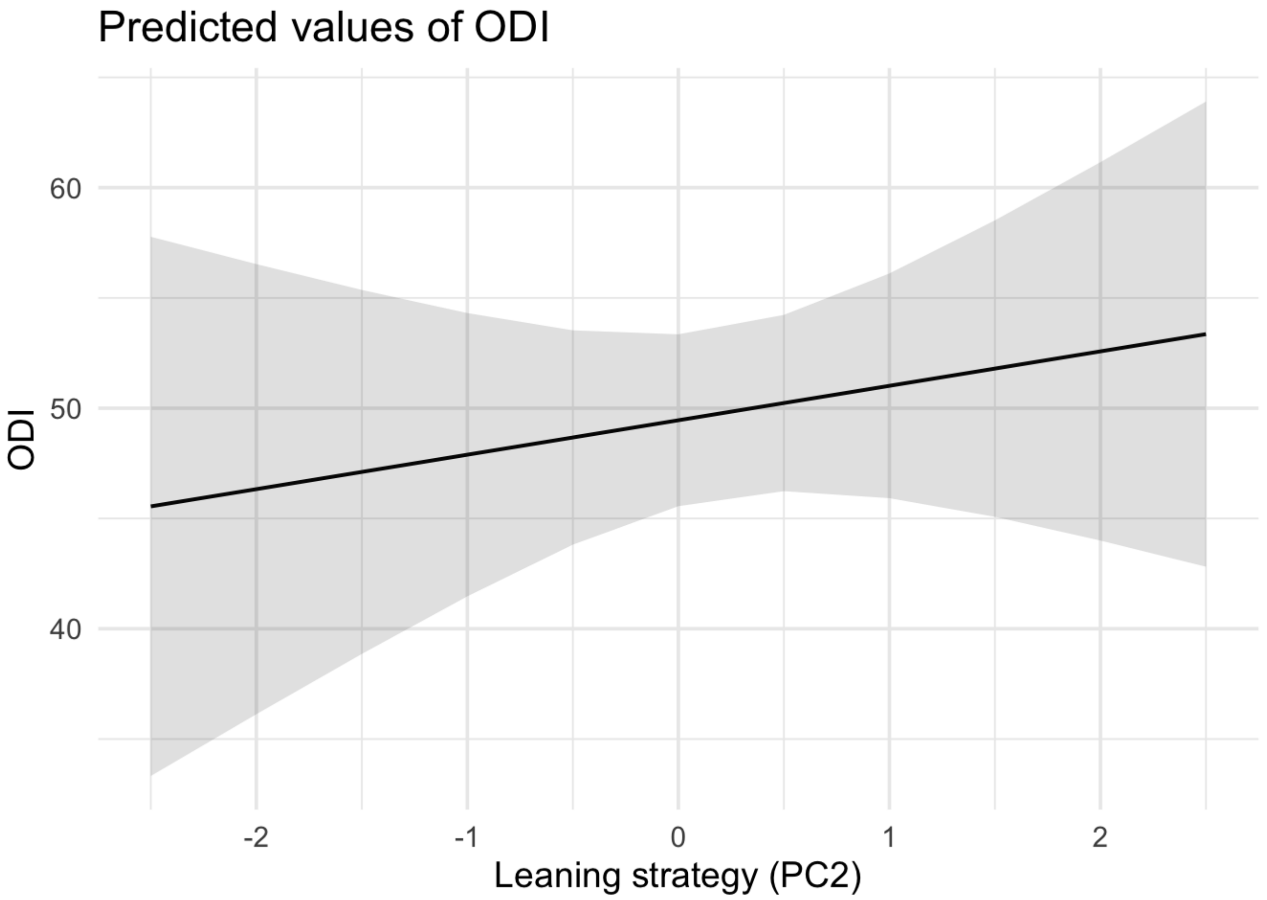

10.6 Fig. 3f

(Linear model results)

```
tab_model(VASback_age_bmi_D2, ODI_age_bmi_D2, show.intercept = F, file = "VAS_O
DI_D2_table.html")
```

	VASback			ODI		
Predictors	*Estimates*	*CI*	*p*	*Estimates*	*CI*	*p*
Age.y	-0.02	-0.07 – 0.02	0.245	-0.00	-0.25 – 0.25	0.999
BMI	0.04	-0.11 – 0.19	0.574	0.23	-0.64 – 1.11	0.597
D2	0.47	-0.28 – 1.21	0.214	1.56	-2.80 – 5.93	0.478
Observations	77			74		
R^2 / R^2 adjusted	0.037 / -0.002			0.011 / -0.031		

Back to top

#Supplemental Fig. 3

reported in the text of the results section

10.7 VASback_PC3

```
VASback_D3 <- lm(VASback ~ Age.y + BMI + D3, data = clinicalvars)
summary(VASback_D3)
```

```
##
## Call:
## lm(formula = VASback ~ Age.y + BMI + D3, data = clinicalvars)
##
## Residuals:
##     Min      1Q  Median      3Q     Max
## -5.9347 -2.0288 -0.2157  2.2096  5.0163
##
## Coefficients:
##             Estimate Std. Error t value Pr(>|t|)
## (Intercept)  5.76958    2.48499   2.322    0.023 *
## Age.y       -0.02101    0.02134  -0.984    0.328
## BMI          0.04096    0.07734   0.530    0.598
## D3           0.09648    0.30530   0.316    0.753
## ---
## Signif. codes:  0 '***' 0.001 '**' 0.01 '*' 0.05 '.' 0.1 ' ' 1
##
## Residual standard error: 2.868 on 73 degrees of freedom
##   (34 observations deleted due to missingness)
## Multiple R-squared:  0.01808,    Adjusted R-squared:  -0.02228
## F-statistic: 0.4479 on 3 and 73 DF,  p-value: 0.7195
```

```
VASback_D3_plot <- plot_model(VASback_D3, type = "pre", terms = "D3", show.data
= F)

VASback_D3_plot+
  theme_minimal(base_size = 16)+
  xlab("Defuse construct (PC3)")
```

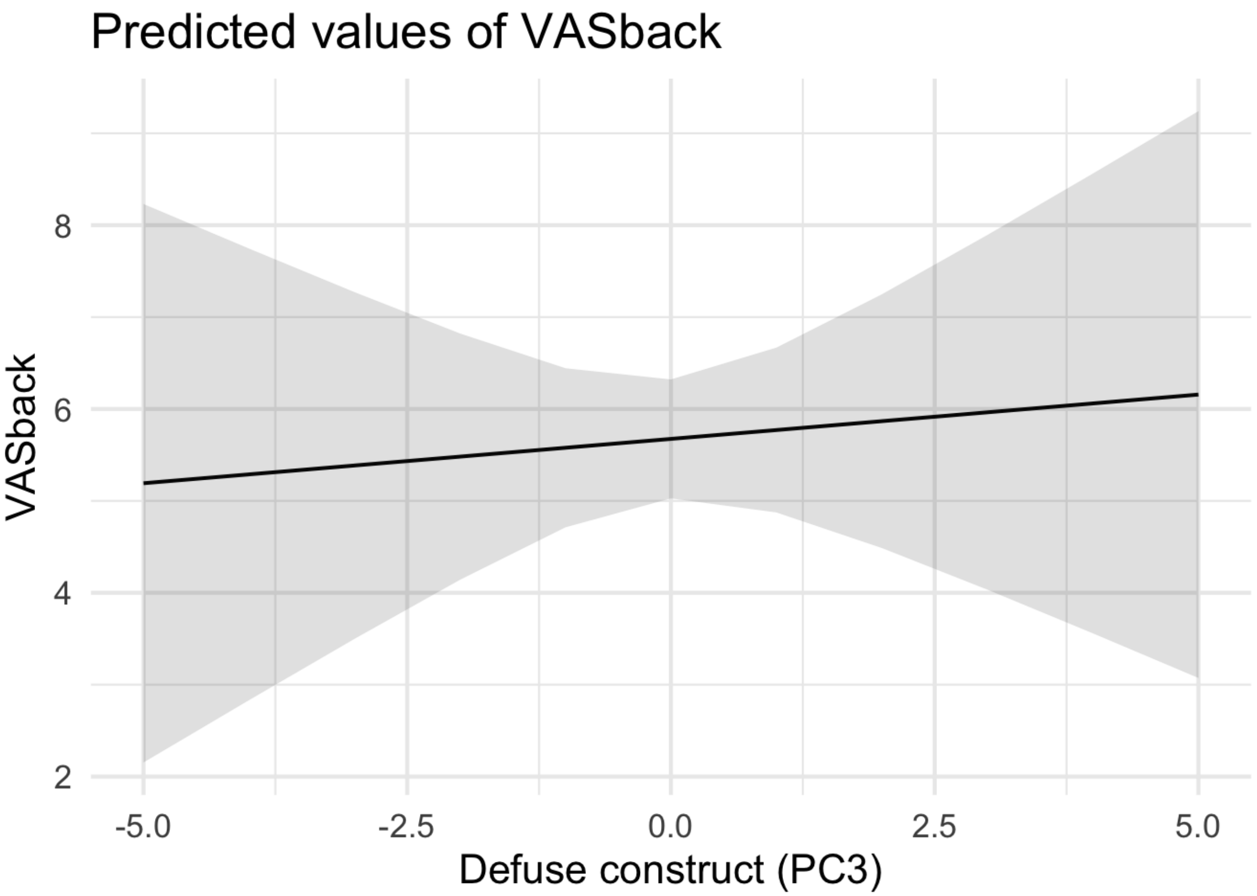

10.8 ODI_PC3

```
ODI_age_bmi_D3 <- lm(ODI ~ Age.y +BMI + D3, data = clinicalvars)
summary(ODI_age_bmi_D3, correlation = T)
```

```
##
## Call:
## lm(formula = ODI ~ Age.y + BMI + D3, data = clinicalvars)
##
## Residuals:
##     Min      1Q  Median      3Q     Max
## -41.872 -11.234   0.087  11.840  40.009
##
## Coefficients:
##             Estimate Std. Error t value Pr(>|t|)
## (Intercept) 42.93388   14.42120   2.977  0.00399 **
## Age.y        0.03437    0.12310   0.279  0.78091
## BMI          0.17768    0.44330   0.401  0.68978
## D3          -1.32253    1.75312  -0.754  0.45315
## ---
## Signif. codes:  0 '***' 0.001 '**' 0.01 '*' 0.05 '.' 0.1 ' ' 1
##
## Residual standard error: 16.4 on 70 degrees of freedom
##   (37 observations deleted due to missingness)
## Multiple R-squared:  0.01191,    Adjusted R-squared:  -0.03044
## F-statistic: 0.2812 on 3 and 70 DF,  p-value: 0.8388
##
## Correlation of Coefficients:
##       (Intercept) Age.y BMI
## Age.y -0.58
## BMI   -0.86        0.12
## D3    -0.03       -0.18  0.15
```

```
ODI_age_bmi_D3_plot <- plot_model(ODI_age_bmi_D3, type = "pre", terms = "D3", s
how.data = F)
ODI_age_bmi_D3_plot +
  theme_minimal(base_size = 14)+
  xlab("Defuse construct (PC3)")
```

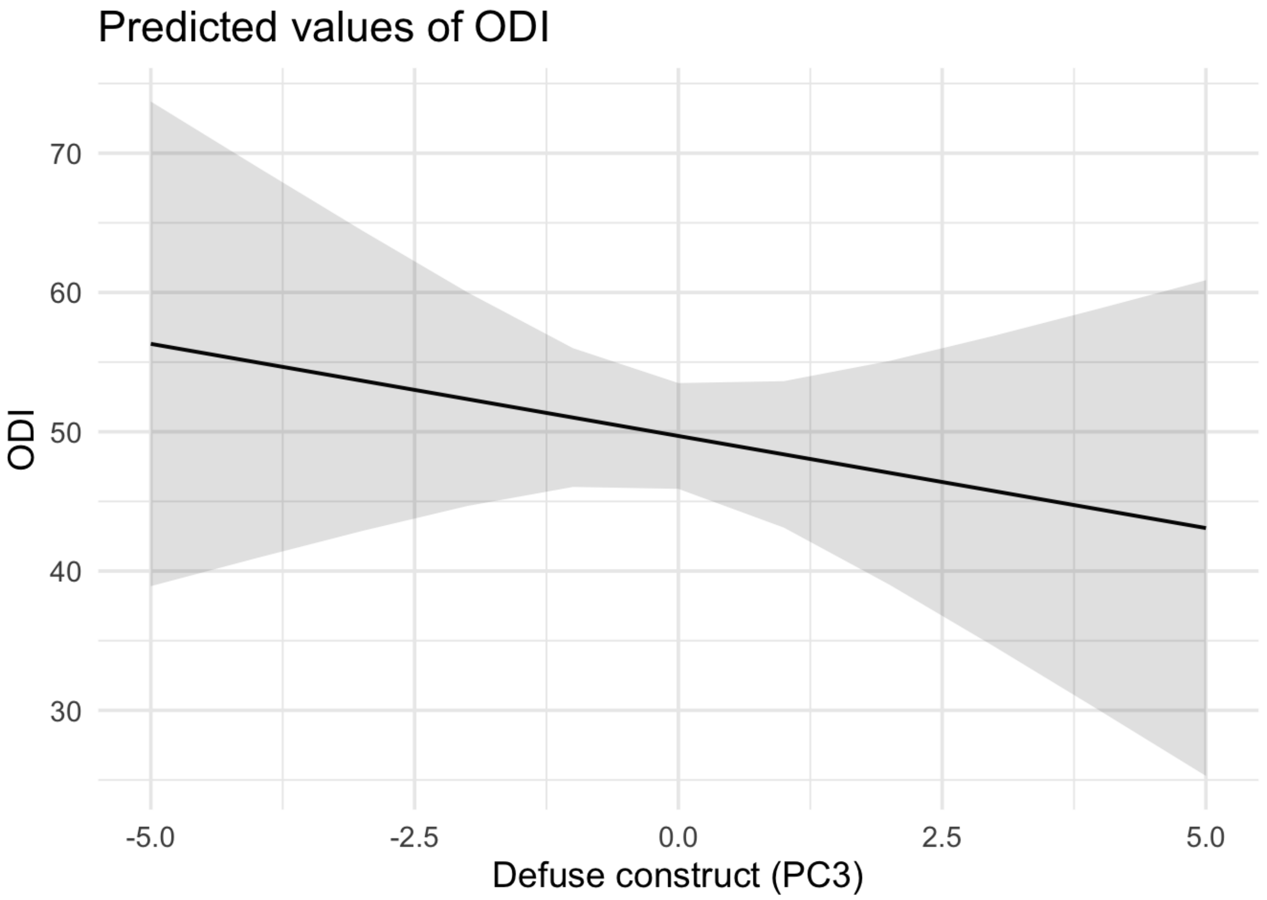

10.9 VASback_ODI_PC3 results

```
tab_model(VASback_D3, ODI_age_bmi_D3, show.intercept = F, file = "VAS_ODI_D3_ta
ble.html")
```

	VASback			ODI		
Predictors	*Estimates*	*CI*	*p*	*Estimates*	*CI*	*p*
Age.y	-0.02	-0.06 – 0.02	0.328	0.03	-0.21 – 0.28	0.781
BMI	0.04	-0.11 – 0.20	0.598	0.18	-0.71 – 1.06	0.690
D3	0.10	-0.51 – 0.70	0.753	-1.32	-4.82 – 2.17	0.453
Observations	77			74		
R^2 / R^2 adjusted	0.018 / -0.022			0.012 / -0.030		

Back to top

11 Table1 Demographics

Note labels: Back pain - NS-LBP, Surgery - SD-LBP

```
table1_demog <- clinicalvars

label(table1_demog$VASback) <- "VAS"
units(table1_demog$Age.y) <- "years"

table1_d <- table1(~Age.y + Sex + BMI + VASback + ODI |Group.x, data = table1_d
emog)

table1(~Age.y + Sex + BMI + VASback + ODI |Group.x, data = table1_demog, overal
l="Total")
```

	Back Pain (N=43)	Control (N=26)	Surgery (N=42)	Total (N=111)
**Age.y (years)**				
Mean (SD)	53.9 (17.4)	27.5 (8.85)	62.7 (11.9)	51.0 (19.3)
Median [Min, Max]	54.0 [21.0, 85.0]	24.0 [18.0, 58.0]	64.5 [30.0, 80.0]	55.0 [18.0, 85.0]
**Sex**				
Mean (SD)	0.558 (0.502)	0.500 (0.510)	0.310 (0.468)	0.450 (0.500)
Median [Min, Max]	1.00 [0, 1.00]	0.500 [0, 1.00]	0 [0, 1.00]	0 [0, 1.00]
**BMI**				
Mean (SD)	26.7 (4.18)	23.8 (3.85)	26.4 (4.86)	25.9 (4.49)
Median [Min, Max]	27.0 [20.0, 41.0]	23.0 [18.0, 33.0]	27.0 [17.0, 38.0]	26.0 [17.0, 41.0]
**VAS**				
Mean (SD)	4.67 (2.38)	NA (NA)	6.83 (2.93)	5.65 (2.84)
Median [Min, Max]	5.00 [0, 9.00]	NA [NA, NA]	8.00 [1.00, 10.0]	6.00 [0, 10.0]
Missing	1 (2.3%)	26 (100%)	7 (16.7%)	34 (30.6%)
**ODI**				
Mean (SD)	50.2 (16.3)	NA (NA)	48.9 (16.2)	49.6 (16.2)
Median [Min, Max]	48.0 [20.0, 88.0]	NA [NA, NA]	52.0 [8.00, 78.0]	50.0 [8.00, 88.0]
Missing	2 (4.7%)	26 (100%)	9 (21.4%)	37 (33.3%)

11.1 Table 1 stats

ANOVAs on demographics (results reported under Table 1)

11.2 ANOVA + posthoc on age by groups

```
ANOVA_Age <-aov(Age.y ~Group.x, data = clinicalvars)
anova(ANOVA_Age)
```

```
## Analysis of Variance Table
##
## Response: Age.y
##             Df Sum Sq Mean Sq F value    Pr(>F)
## Group.x      2  20544 10272.1  54.133 < 2.2e-16 ***
## Residuals 108  20494   189.8
## ---
## Signif. codes:  0 '***' 0.001 '**' 0.01 '*' 0.05 '.' 0.1 ' ' 1
```

```
tukeyresults_Age <- TukeyHSD(x= ANOVA_Age)
TukeyHSD(x= ANOVA_Age)
```

```
##   Tukey multiple comparisons of means
##     95% family-wise confidence level
##
## Fit: aov(formula = Age.y ~ Group.x, data = clinicalvars)
##
## $Group.x
##                          diff        lwr       upr     p adj
## Control-Back Pain -26.39893 -34.531586 -18.26627 0.0000000
## Surgery-Back Pain   8.87763   1.775649  15.97961 0.0101639
## Surgery-Control    35.27656  27.107497  43.44562 0.0000000
```

Back to top

11.3 ANOVA + posthoc on BMI by groups

```
ANOVA_BMI <-aov(BMI ~Group.x, data = clinicalvars)
anova(ANOVA_BMI)
```

```
## Analysis of Variance Table
##
## Response: BMI
##             Df  Sum Sq Mean Sq F value  Pr(>F)
## Group.x      2  147.16  73.579  3.8294 0.02473 *
## Residuals 108 2075.11  19.214
## ---
## Signif. codes:  0 '***' 0.001 '**' 0.01 '*' 0.05 '.' 0.1 ' ' 1
```

```
tukeyresults_BMI <-TukeyHSD(x = ANOVA_BMI)
TukeyHSD (x = ANOVA_BMI)
```

```
##   Tukey multiple comparisons of means
##     95% family-wise confidence level
##
## Fit: aov(formula = BMI ~ Group.x, data = clinicalvars)
##
## $Group.x
##                          diff         lwr        upr     p adj
## Control-Back Pain -2.8282648 -5.41613646 -0.2403931 0.0286336
## Surgery-Back Pain -0.2458472 -2.50574960  2.0140552 0.9638392
## Surgery-Control    2.5824176 -0.01703701  5.1818722 0.0519226
```

11.4 ANOVA + posthoc on VAS scores by groups

```
ANOVA_VAS <-aov(VASback ~Group.x, data = clinicalvars)
anova(ANOVA_VAS)
```

```
## Analysis of Variance Table
##
## Response: VASback
##           Df Sum Sq Mean Sq F value   Pr(>F)
## Group.x    1  89.23  89.228  12.813 0.000608 ***
## Residuals 75 522.30   6.964
## ---
## Signif. codes:  0 '***' 0.001 '**' 0.01 '*' 0.05 '.' 0.1 ' ' 1
```

```
tukeyresults_VAS <-TukeyHSD(x = ANOVA_VAS)
TukeyHSD (x = ANOVA_VAS)
```

```
##   Tukey multiple comparisons of means
##     95% family-wise confidence level
##
## Fit: aov(formula = VASback ~ Group.x, data = clinicalvars)
##
## $Group.x
##                        diff       lwr      upr    p adj
## Surgery-Back Pain 2.161905 0.9587274 3.365082 0.000608
```

11.5 ANOVA on ODI scores by groups

```
ANOVA_ODI <-aov(ODI ~Group.x, data = clinicalvars)
anova(ANOVA_ODI)
```

```
## Analysis of Variance Table
##
## Response: ODI
##           Df  Sum Sq Mean Sq F value Pr(>F)
## Group.x    1    31.7  31.681    0.12 0.7301
## Residuals 72 19014.0 264.083
```

Back to top

11.6 Xsquare test for sex distribution by groups

```
Xsq_Sex <- chisq.test(table(clinicalvars$Group.x, clinicalvars$Sex))
Xsq_Sex
```

```
##
##  Pearson's Chi-squared test
##
## data:  table(clinicalvars$Group.x, clinicalvars$Sex)
## X-squared = 5.642, df = 2, p-value = 0.05955
```

12 PC4-PC7 - supplemental material

```
ave_trialsT_loadingsD4 <-stand_loadings(ave_trialsT_nlpca, ave_trials_transform
ed)%>%
  arrange(PC4)%>%
  #select(PC1,PC2,PC3)%>%
  filter(abs(PC4)>=0.5)

heatmap_loading(ave_trialsT_nlpca, ave_trials_transformed, ndim = 4, text_value
s = T, star_values = F, vars = rownames(ave_trialsT_loadingsD4))
```

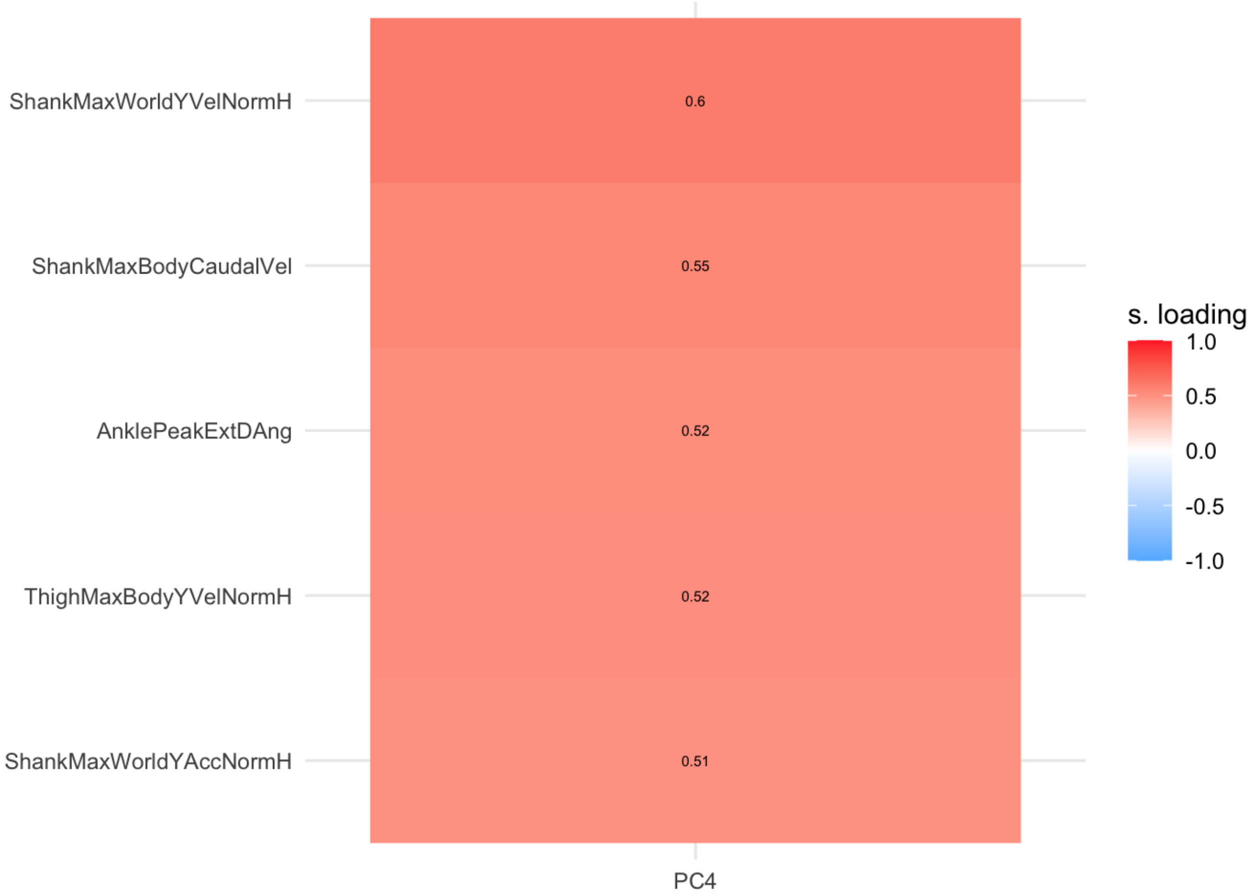


```
ave_trialsT_loadingsD5 <-stand_loadings(ave_trialsT_nlpca, ave_trials_transform
ed)%>%
  arrange(PC5)%>%
  #select(PC1,PC2,PC3)%>%
  filter(abs(PC5)>=0.5)

heatmap_loading(ave_trialsT_nlpca, ave_trials_transformed, ndim = 5, text_value
s = T, star_values = F, vars = rownames(ave_trialsT_loadingsD5))
```

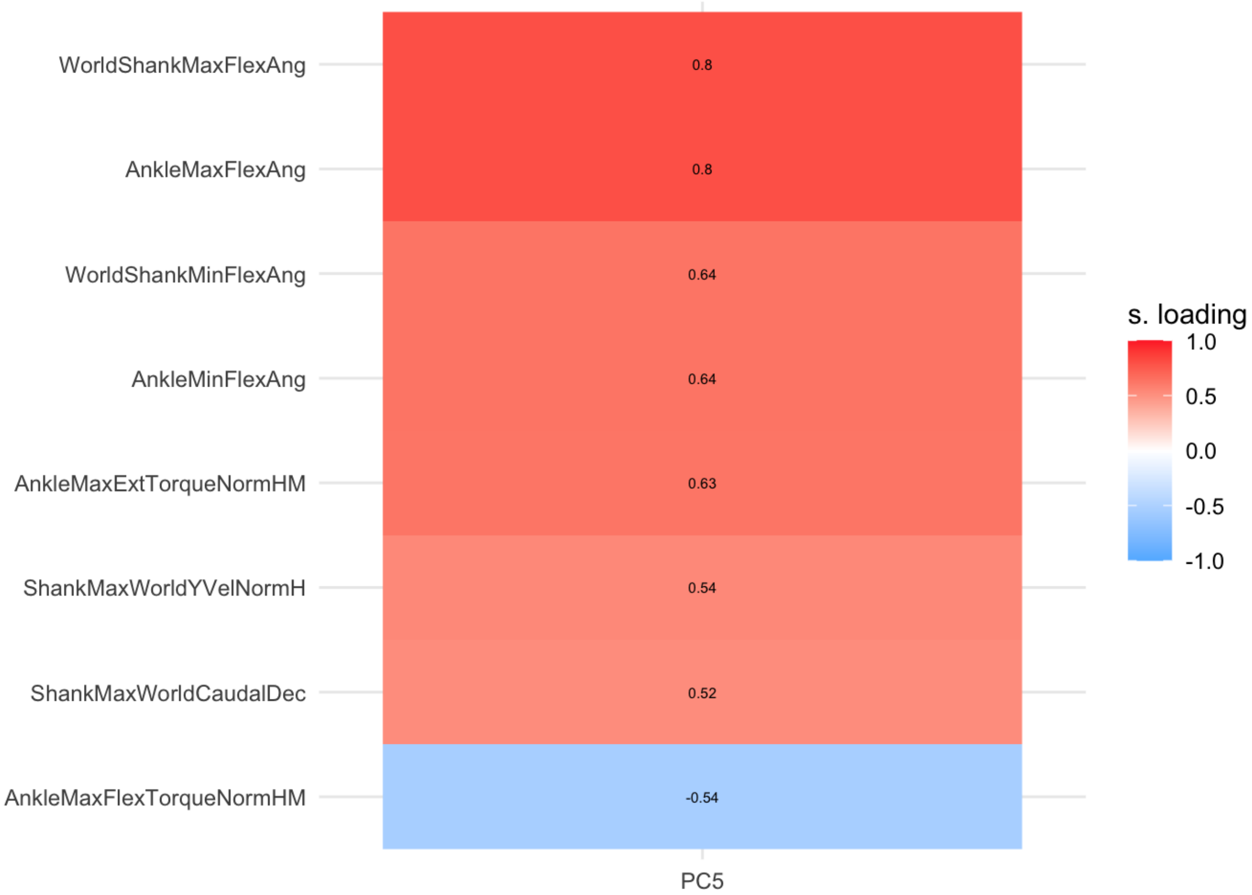


```
ave_trialsT_loadingsD6 <-stand_loadings(ave_trialsT_nlpca, ave_trials_transform
ed)%>%
  arrange(PC6)%>%
  #select(PC1,PC2,PC3)%>%
  filter(abs(PC6)>=0.5)

heatmap_loading(ave_trialsT_nlpca, ave_trials_transformed, ndim = 6, text_value
s = T, star_values = F, vars = rownames(ave_trialsT_loadingsD6))
```

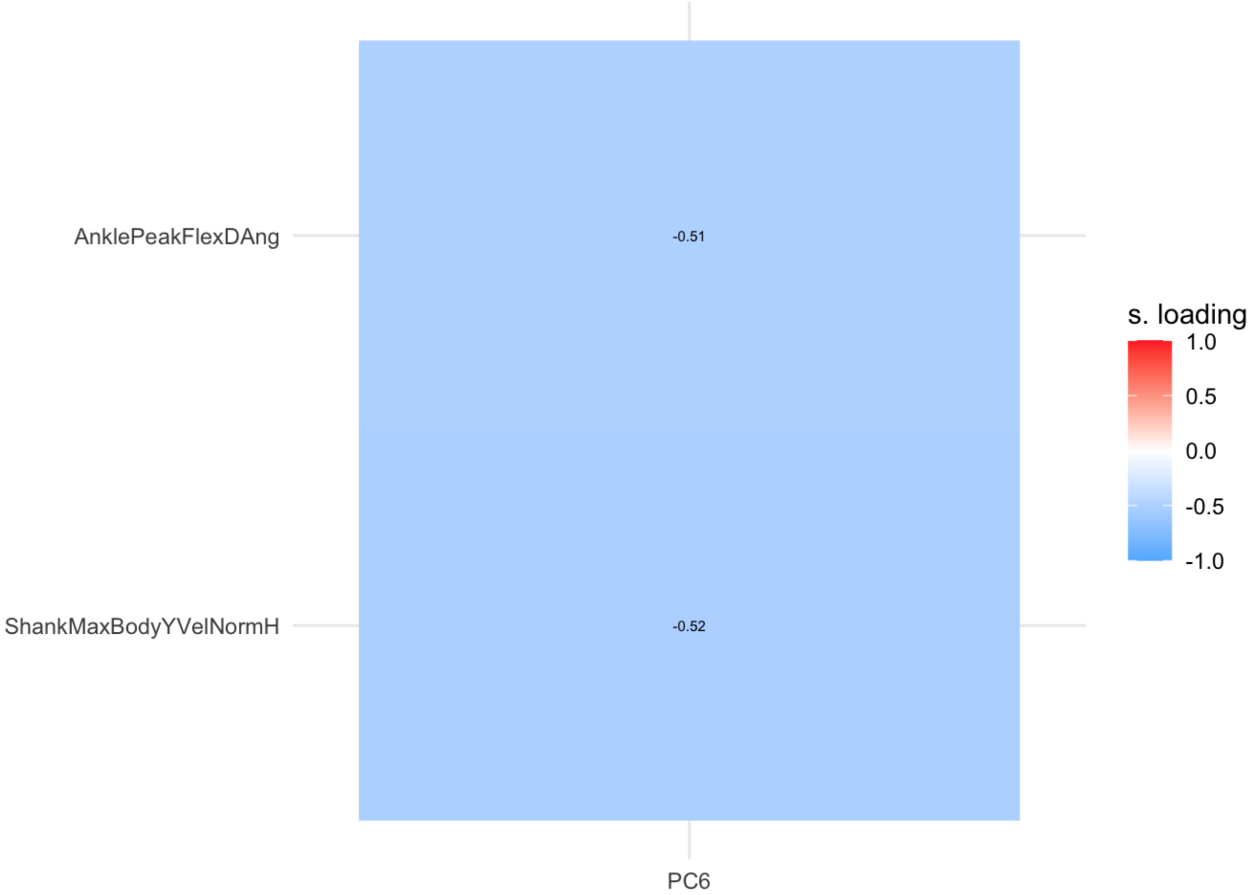


```
ave_trialsT_loadingsD7 <-stand_loadings(ave_trialsT_nlpca, ave_trials_transform
ed)%>%
  arrange(PC7)%>%
  #select(PC1,PC2,PC3)%>%
  filter(abs(PC7)>=0.5)

PC7heatmap <- heatmap_loading(ave_trialsT_nlpca, ave_trials_transformed, ndim =
7, text_values = FALSE, star_values = T, vars = rownames(ave_trialsT_loadingsD
7))

# no loadings above the cut off of 0.5
```

Back to top

13 Supplemtanl Fig. 4 (PC4-PC6 bar plots) + ANOVA tests

NS = not significant

```
ggplot(as.data.frame(ave_trialsT_nlpca$objectscores), aes(x=groups, y = D4, fil
l=groups))+
  geom_boxplot()+
  geom_point()+
  theme_minimal(base_size = 25)+
  xlab(NULL)+
  ylab("PC4")+
  #ylim(c(-15,15))+
  labs(fill=NULL)+
  scale_x_discrete(breaks=c(3,2,1))+
  theme(legend.position=c(.9,.75))+
  theme(legend.position = "left bottom",legend.text = element_text(size = 12))+
  theme (legend.title = element_blank())+
  scale_fill_manual(values = c("black", "blue3", "brown2"))
```

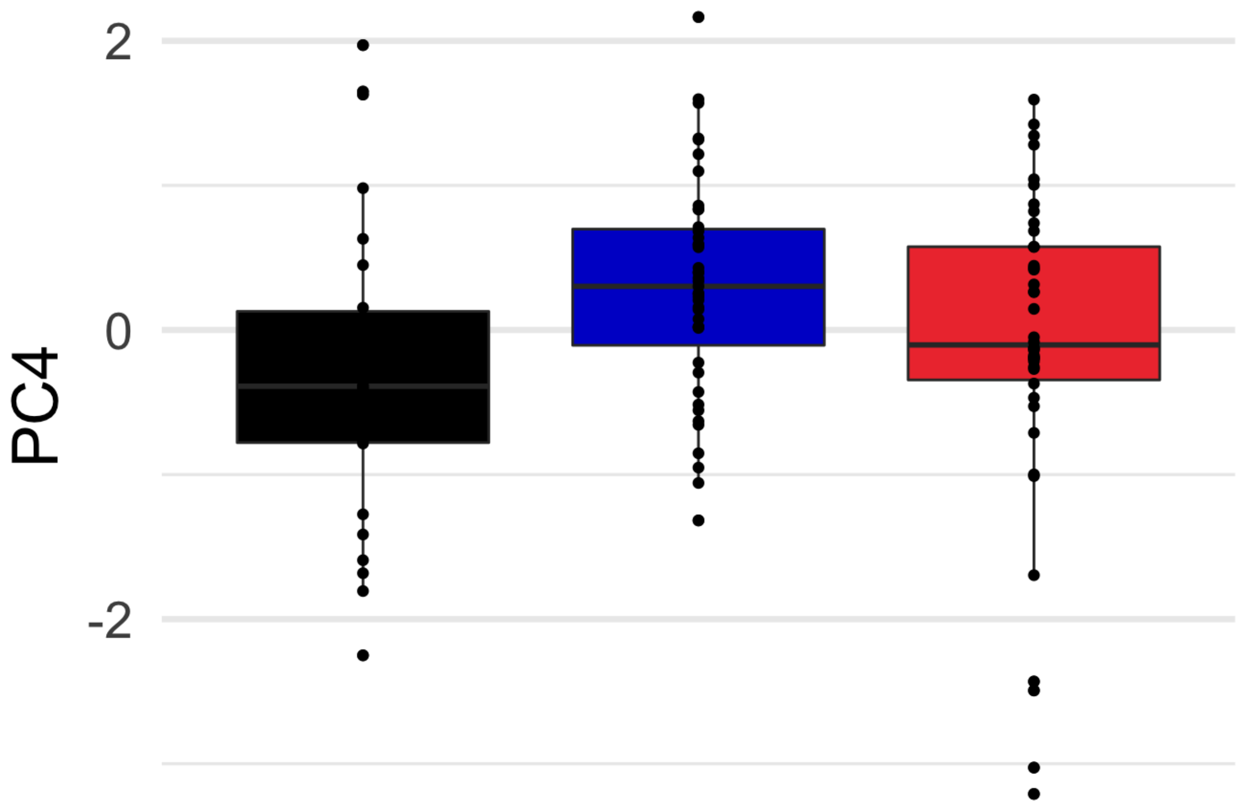


```
ANOVA_aveT_nlpca_PC4 <-aov(D4~groups, data = as.data.frame(ave_trialsT_nlpca$ob
jectscores))
anova(ANOVA_aveT_nlpca_PC4)
```

```
## Analysis of Variance Table
##
## Response: D4
##            Df  Sum Sq Mean Sq F value  Pr(>F)
## groups      2   6.732  3.3661  3.4866 0.03409 *
## Residuals 108 104.268  0.9654
## ---
## Signif. codes:  0 '***' 0.001 '**' 0.01 '*' 0.05 '.' 0.1 ' ' 1
```

```
tukeyresults_aveT_PC4 <- TukeyHSD(x= ANOVA_aveT_nlpca_PC4)
TukeyHSD(x= ANOVA_aveT_nlpca_PC4)
```

```
##   Tukey multiple comparisons of means
##     95% family-wise confidence level
##
## Fit: aov(formula = D4 ~ groups, data = as.data.frame(ave_trialsT_nlpca$objec
tscores))
##
## $groups
##                       diff         lwr        upr     p adj
## NS-LBP-Control  0.6019995  0.02190712 1.18209194 0.0400637
## SD-LBP-Control  0.1936493 -0.38903950 0.77633812 0.7100958
## SD-LBP-NS-LBP  -0.4083502 -0.91492565 0.09822522 0.1391643
```

```
ggplot(as.data.frame(ave_trialsT_nlpca$objectscores), aes(x=groups, y = D5, fil
l=groups))+
  geom_boxplot()+
  geom_point()+
  theme_minimal(base_size = 25)+
  xlab(NULL)+
  ylab("PC5")+
  #ylim(c(-15,15))+
  labs(fill=NULL)+
  scale_x_discrete(breaks=c(3,2,1))+
  theme(legend.position=c(.9,.75))+
  theme(legend.position = "left bottom",legend.text = element_text(size = 12))+
  theme (legend.title = element_blank())+
  scale_fill_manual(values = c("black", "blue3", "brown2"))
```

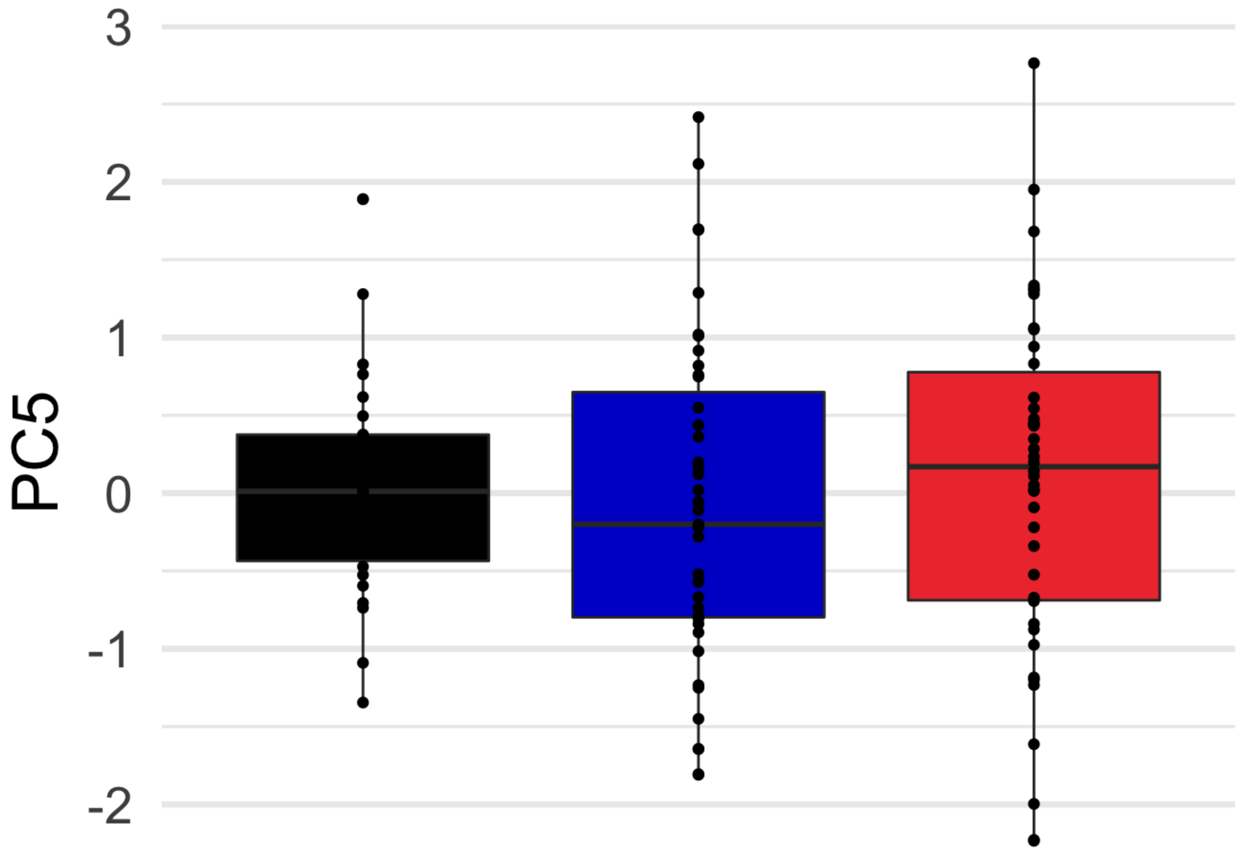


```
ANOVA_aveT_nlpca_PC5 <-aov(D5~groups, data = as.data.frame(ave_trialsT_nlpca$ob
jectscores))
anova(ANOVA_aveT_nlpca_PC5)
```

```
## Analysis of Variance Table
##
## Response: D5
##            Df  Sum Sq Mean Sq F value Pr(>F)
## groups      2   0.586 0.29291  0.2865 0.7515
## Residuals 108 110.414 1.02235
```

```
#NS

ggplot(as.data.frame(ave_trialsT_nlpca$objectscores), aes(x=groups, y = D6, fil
l=groups))+
  geom_boxplot()+
  geom_point()+
  theme_minimal(base_size = 25)+
  xlab(NULL)+
  ylab("PC6")+
  #ylim(c(-15,15))+
  labs(fill=NULL)+
  scale_x_discrete(breaks=c(3,2,1))+
  theme(legend.position=c(.9,.75))+
  theme(legend.position = "left bottom",legend.text = element_text(size = 12))+
  theme (legend.title = element_blank())+
  scale_fill_manual(values = c("black", "blue3", "brown2"))
```

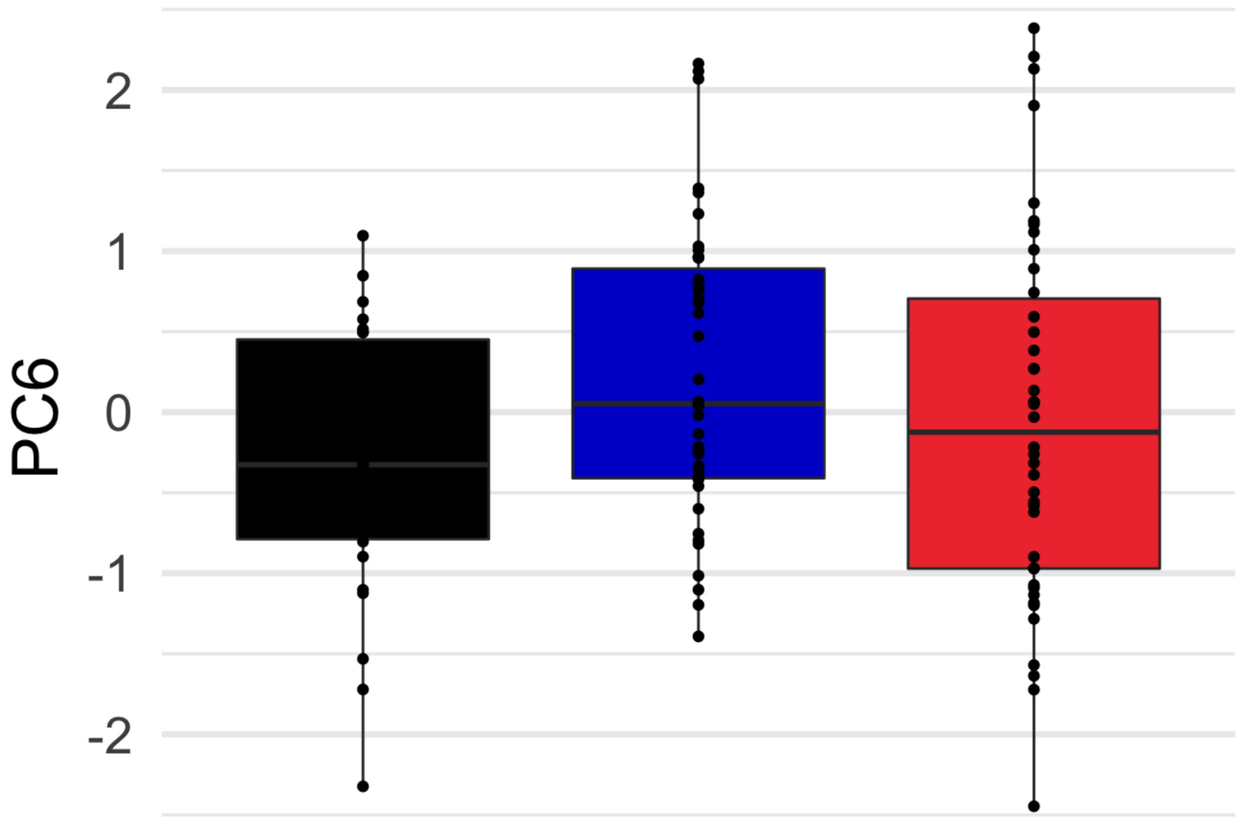


```
ANOVA_aveT_nlpca_PC6 <-aov(D6~groups, data = as.data.frame(ave_trialsT_nlpca$ob
jectscores))
anova(ANOVA_aveT_nlpca_PC6)
```

```
## Analysis of Variance Table
##
## Response: D6
##             Df  Sum Sq Mean Sq F value  Pr(>F)
## groups       2   4.793  2.3964  2.4369 0.09223 .
## Residuals  108 106.207  0.9834
## ---
## Signif. codes:  0 '***' 0.001 '**' 0.01 '*' 0.05 '.' 0.1 ' ' 1
```

```
#NS
```

Back to top

14 Session info

```
sessionInfo()
```

```
## R version 4.0.2 (2020-06-22)
## Platform: x86_64-apple-darwin17.0 (64-bit)
## Running under: macOS  10.16
##
## Matrix products: default
## BLAS:   /Library/Frameworks/R.framework/Versions/4.0/Resources/lib/libRblas.
dylib
## LAPACK: /Library/Frameworks/R.framework/Versions/4.0/Resources/lib/libRlapac
k.dylib
##
## locale:
## [1] en_US.UTF-8/en_US.UTF-8/en_US.UTF-8/C/en_US.UTF-8/en_US.UTF-8
##
## attached base packages:
## [1] stats     graphics  grDevices utils     datasets  methods   base
##
## other attached packages:
##  [1] tableHTML_2.1.0      knitr_1.29           table1_1.4.2
##  [4] sjPlot_2.8.9         ISLR_1.2             psych_2.0.12
##  [7] Gifi_0.3-9           car_3.0-10           carData_3.0-4
## [10] scatterplot3d_0.3-41 plotly_4.9.2.1       syndRomics_0.1.0
## [13] progress_1.2.2       forcats_0.5.0        stringr_1.4.0
## [16] dplyr_1.0.7          purrr_0.3.4          readr_1.3.1
## [19] tidyr_1.1.3          tibble_3.1.2         ggplot2_3.3.5
## [22] tidyverse_1.3.0
##
## loaded via a namespace (and not attached):
##  [1] minqa_1.2.4       ggnewscale_0.4.5  colorspace_2.0-2  ellipsis_0.3.2
##  [5] rio_0.5.16        sjlabelled_1.1.8  estimability_1.3  parameters_0.14.0
##  [9] fs_1.5.0          rstudioapi_0.11   farver_2.1.0      ggrepel_0.9.1
## [13] fansi_0.5.0       mvtnorm_1.1-2     lubridate_1.7.9   xml2_1.3.2
## [17] codetools_0.2-16  splines_4.0.2     mnormt_2.0.2      sjmisc_2.8.7
## [21] Formula_1.2-4     jsonlite_1.7.0    nloptr_1.2.2.2    ggeffects_1.1.1
## [25] broom_0.7.0       dbplyr_1.4.4      effectsize_0.4.5  compiler_4.0.2
## [29] httr_1.4.2        sjstats_0.18.1    emmeans_1.6.2-1   backports_1.1.8
## [33] Matrix_1.2-18     assertthat_0.2.1  lazyeval_0.2.2    cli_3.0.1
## [37] htmltools_0.5.0   prettyunits_1.1.1 tools_4.0.2       coda_0.19-3
## [41] gtable_0.3.0      glue_1.4.2        Rcpp_1.0.7        cellranger_1.1.0
## [45] vctrs_0.3.8       nlme_3.1-148      insight_0.14.2    xfun_0.16
## [49] openxlsx_4.2.3    lme4_1.1-26       rvest_0.3.6       lifecycle_1.0.0
## [53] statmod_1.4.35    MASS_7.3-51.6     scales_1.1.1      hms_1.1.0
## [57] parallel_4.0.2    yaml_2.2.1        curl_4.3          pbapply_1.4-3
## [61] stringi_1.7.3     bayestestR_0.10.5 boot_1.3-25       zip_2.1.1
## [65] rlang_0.4.11      pkgconfig_2.0.3   pracma_2.3.3      evaluate_0.14
## [69] lattice_0.20-41   labeling_0.4.2    htmlwidgets_1.5.1 tidyselect_1.1.1
## [73] magrittr_2.0.1    R6_2.5.0          generics_0.1.0    DBI_1.1.0
## [77] pillar_1.6.1      haven_2.3.1       foreign_0.8-80    withr_2.4.2
## [81] datawizard_0.1.0  abind_1.4-5       performance_0.7.3 modelr_0.1.8
## [85] crayon_1.4.1      utf8_1.2.1        tmvnsim_1.0-2     rmarkdown_2.3
## [89] grid_4.0.2        readxl_1.3.1      data.table_1.13.2 blob_1.2.1
## [93] reprex_0.3.0      digest_0.6.27     xtable_1.8-4      munsell_0.5.0
## [97] viridisLite_0.4.0
```

Back to top
