# Supplementary material for: Unsupervised Machine Learning on Motion Capture Data Uncovers Movement Strategies in Low Back Pain
Source: Front Bioeng Biotechnol. 2022 Apr 14;10:868684. doi: 10.3389/fbioe.2022.868684 (PMC9047543; doi:10.3389/fbioe.2022.868684)
Supplement: Supplementary file 2 [file DataSheet1.PDF]

Supplemental File 1. Data Dictionary (page 1-10)

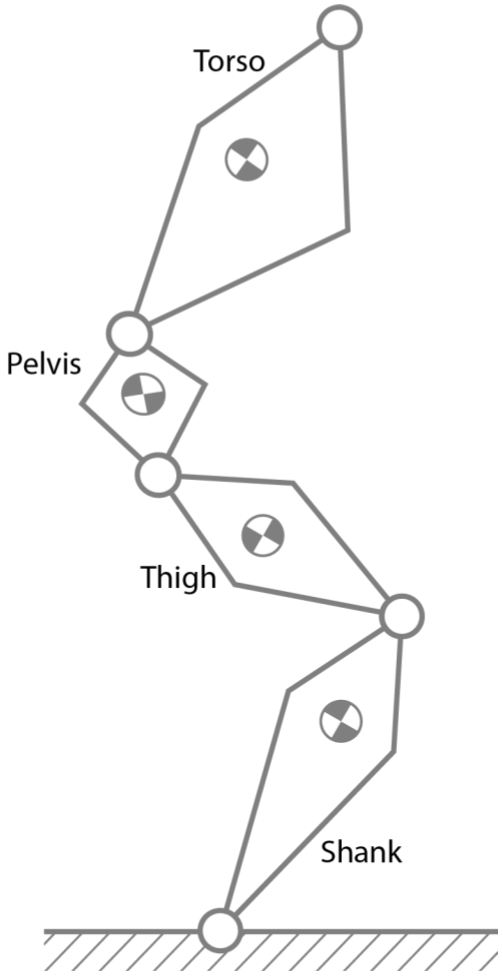

1 Primary Measures

Primary Measures are restricted to Kinematic joint angles and positions that are obtained directly from the sensor or can be obtained from through direct computation

1.1 Relative Joint Angles

ID	Variant	Units	Description
Ankle Flex Angle	Max, Min	Degrees	Relative flexion angle between the world frame vertical and the shank.
Knee Flex Angle	Max, Min	Degrees	Relative flexion angle between the shank and the thigh.
Hip Flex Angle	Max, Min	Degrees	Relative flexion angle between the thigh and the pelvis.
L5S1 Flex Angle	Max, Min	Degrees	Relative flexion angle between the pelvis and the torso.

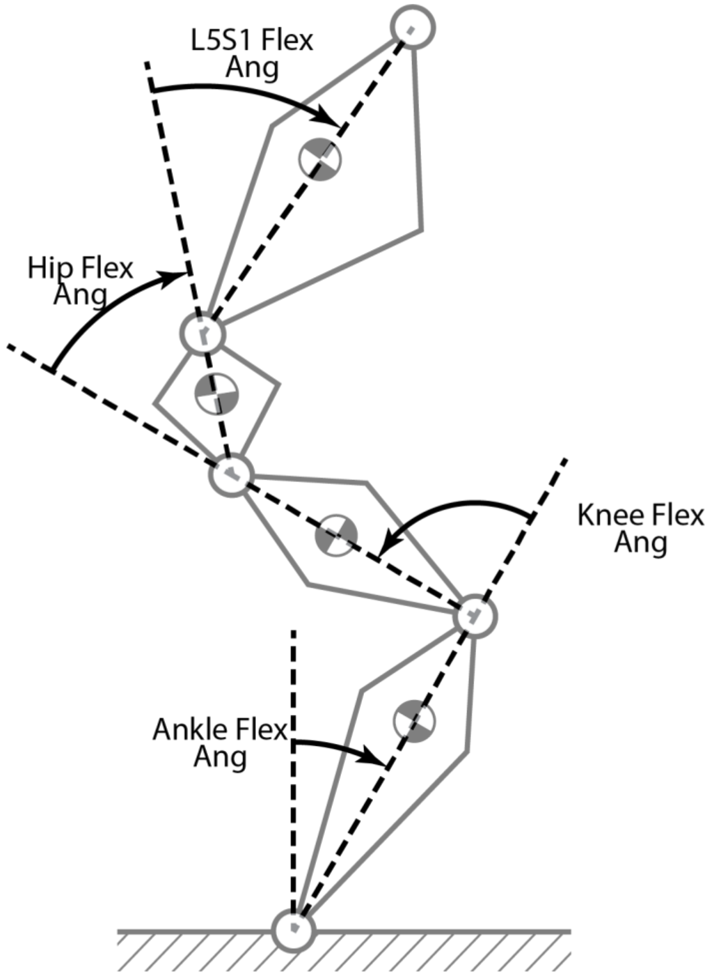

1.2 World Segment Angles

ID	Variant	Units	Description
World Ankle Flex Angle	Max, Min	Degrees	World frame angle between the world-frame vertical and the shank.
World Knee Flex Angle	Max, Min	Degrees	World frame angle between the world-frame vertical and the thigh.
World Hip Flex Angle	Max, Min	Degrees	World frame angle between the world-frame vertical and the pelvis.
World L5S1 Flex Angle	Max, Min	Degrees	World frame angle between the world-frame vertical and the torso.

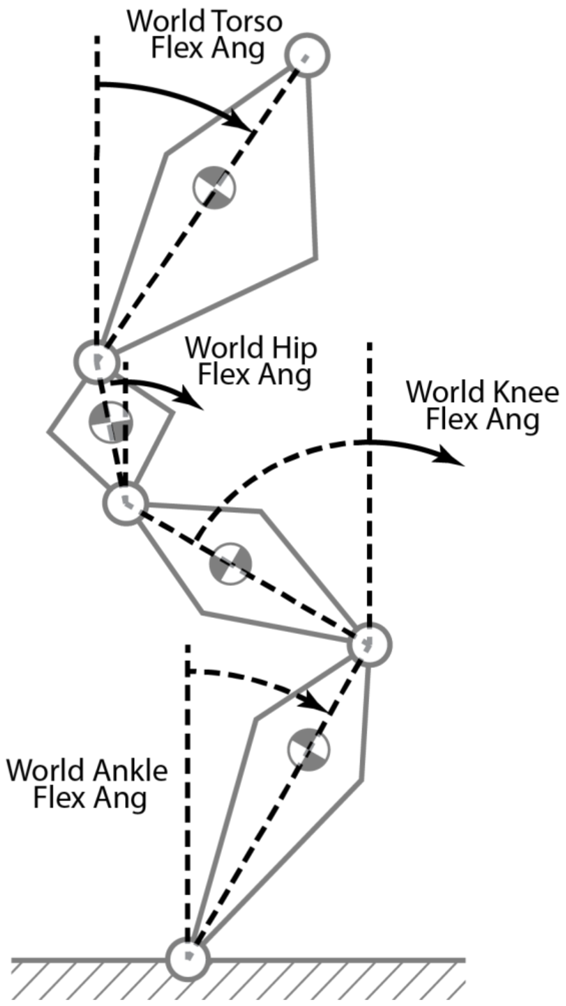

1.3 Sagittal Vertical Axis

ID	Variant	Units	Description
SVA	Max	Meters	The maximum distance between the hip centre and shoulder centre, when projected into the ground plane. As this study is limited to movements in the sagittal plane, this is the maximum difference in the x-coordinates of the shoulder and hip. This measure captures the how far the subject leans forward during the standing action
SVA Norm H	Max	none	SVA normalized by subject height

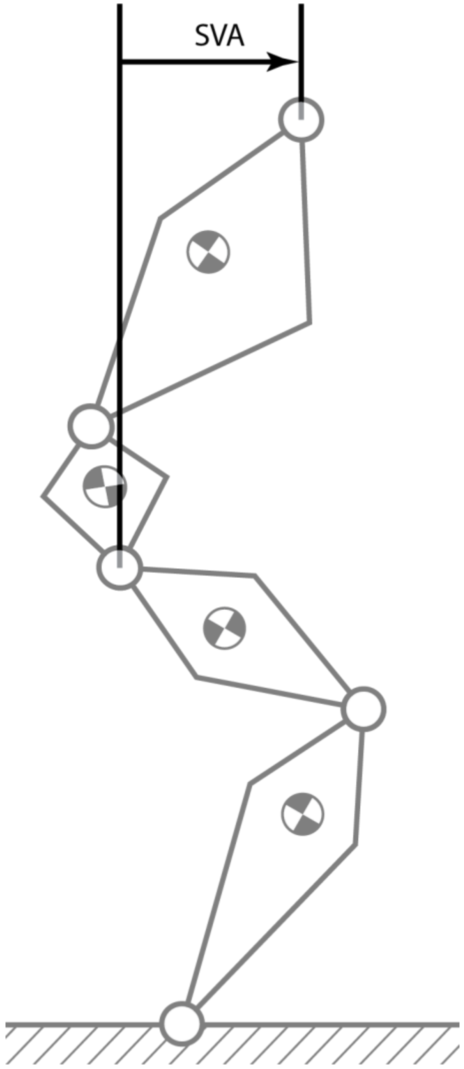

2 Secondary Measures

Kinetic measures obtained from the primary measures. Kinetics are obtained through

Normalisation by time is based on the Time from static sitting to static standing.

2.1 Relative Joint Angular Velocities and Accelerations

ID	Variant	Units	Description
Ankle Flex DAngle	Flex, Ext	Deg/s	Relative flexion angular velocity between the world frame vertical and the shank.
Knee Flex DAngle	Flex, Ext	Deg/s	Relative flexion angular velocity between the shank and the thigh.
Hip Flex DAngle	Flex, Ext	Deg/s	Relative flexion angular velocity between the thigh and the pelvis.
L5S1 Flex DAngle	Flex, Ext	Deg/s	Relative flexion angular velocity between the pelvis and the torso.
Ankle Flex DDAngle	Flex, Ext	Deg/s^2	Relative flexion angular acceleration between the world frame vertical and the shank.
Knee Flex DDAngle	Flex, Ext	Deg/s^2	Relative flexion angular acceleration between the shank and the thigh.
Hip Flex DDAngle	Flex, Ext	Deg/s^2	Relative flexion angular acceleration between the thigh and the pelvis.
L5S1 Flex DDAngle	Flex, Ext	Deg/s^2	Relative flexion angular acceleration between the pelvis and the torso.

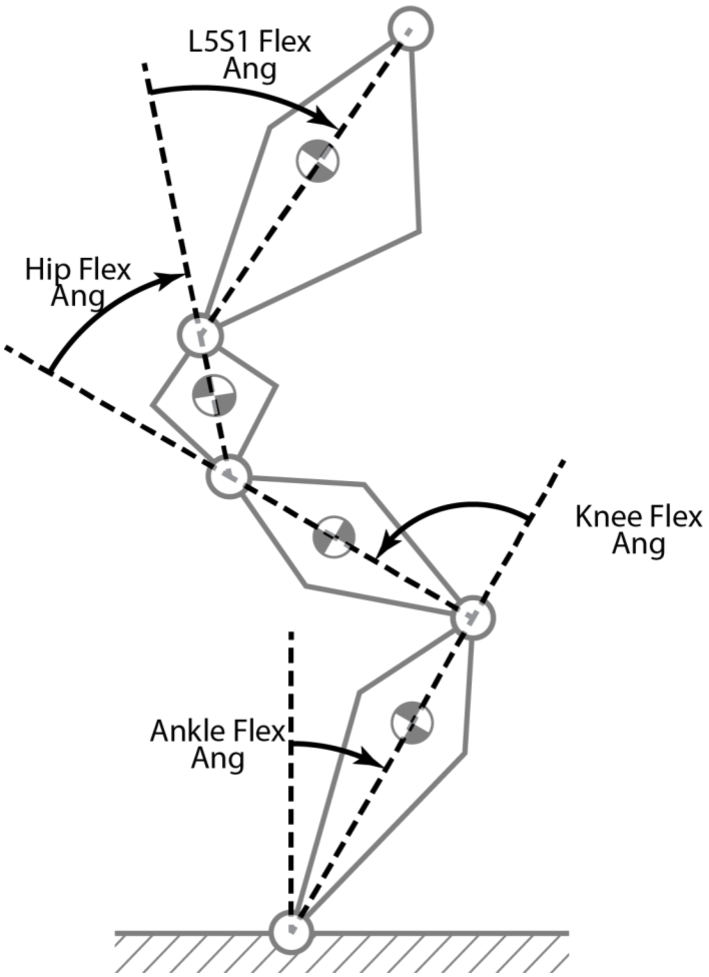

2.2 Body Segment Velocities and Accelerations

ID	Variant	Units	Description
Shank Vel	X, Y	m/s	Shank body velocity written in the shank coordinate frame
Thigh Vel	X, Y	m/s	Thigh body velocity written in the thigh coordinate frame
Pelvis Vel	X, Y	m/s	Pelvis body velocity written in the pelvis coordinate frame
Torso Vel	X, Y	m/s	Torso body velocity written in the toros coordinate frame
Shank Acc	X, Y	m/s^2	Shank body acceleration written in the shank coordinate frame
Thigh Acc	X, Y	m/s^2	Thigh body acceleration written in the thigh coordinate frame
Pelvis Acc	X, Y	m/s^2	Pelvis body acceleration written in the pelvis coordinate frame
Torso Acc	X, Y	m/s^2	Torso body acceleration written in the torso coordinate frame
Shank Vel NormHT	X, Y	-	Normalise by dividing by H and multiplying by T or TT.
Thigh Vel NormHT	X, Y	-	
Pelvis Vel NormHT	X, Y	-	
Torso Vel NormHT	X, Y	-	
Shank Acc NormHTT	X, Y	-	
Thigh Acc NormHTT	X, Y	-	
Pelvis Acc NormHTT	X, Y	-	
Torso Acc NormHTT	X, Y	-	

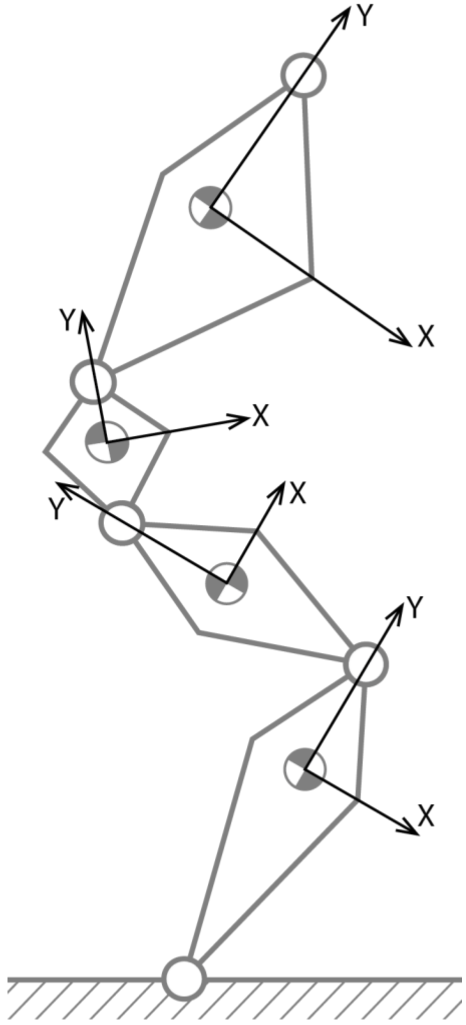

2.3 World Segment Velocities and Accelerations

ID	Variant	Units	Description
Shank Vel	X, Y	m/s	Shank body velocity written in the world coordinate frame
Thigh Vel	X, Y	m/s	Thigh body velocity written in the world coordinate frame
Pelvis Vel	X, Y	m/s	Pelvis body velocity written in the world coordinate frame
Torso Vel	X, Y	m/s	Torso body velocity written in the world coordinate frame
Shank Acc	X, Y	m/s^2	Shank body acceleration written in the world coordinate frame
Thigh Acc	X, Y	m/s^2	Thigh body acceleration written in the world coordinate frame
Pelvis Acc	X, Y	m/s^2	Pelvis body acceleration written in the world coordinate frame
Torso Acc	X, Y	m/s^2	Torso body acceleration written in the world coordinate frame
Shank Vel NormHT	X, Y	-	Normalise by dividing by H and multiplying by T or TT.
Thigh Vel NormHT	X, Y	-	
Pelvis Vel NormHT	X, Y	-	
Torso Vel NormHT	X, Y	-	
Shank Acc NormHTT	X, Y	-	
Thigh Acc NormHTT	X, Y	-	
Pelvis Acc NormHTT	X, Y	-	
Torso Acc NormHTT	X, Y	-	

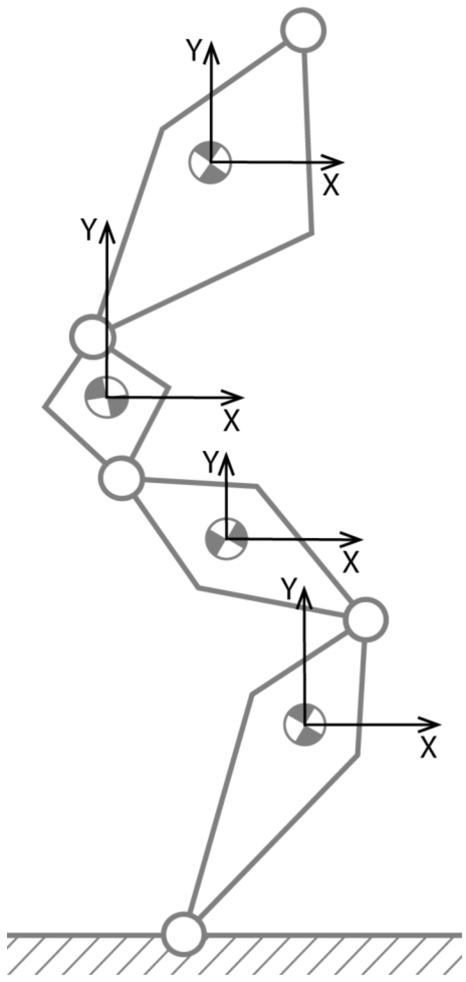

3 Tertiary Measures

Torque (Moment) Normalisation by (Body Mass x Height) is based on the recommendations of:

- Moisio KC, Sumner DR, Shott S, Hurwitz DE. Normalization of joint moments during gait: a comparison of two techniques. Journal of biomechanics. 2003 Apr 1;36(4):599-603.
- Bazett-Jones DM, Cobb SC, Joshi MN, Cashin SE, Earl JE. Normalizing hip muscle strength: establishing body-size-independent measurements. Archives of physical medicine and rehabilitation. 2011 Jan 1;92(1):76-82.
- Pinzone O, Schwartz MH, Baker R. Comprehensive non-dimensional normalization of gait data. Gait & posture. 2016 Feb 1;44:68-73.

3.1 Joint Torques

ID	Variant	Units	Description
**Ankle Flex Torque**	Max, Min	Nm	Computed via Inverse dynamics
**Knee Flex Torque**	Max, Min	Nm	
**Hip Flex Torque**	Max, Min	Nm	
**L5S1 Flex Torque**	Max, Min	Nm	
**Ankle Flex Torque NormHM**	Max, Min	Nm/kgm	Torques normalized by subject Mass x Height
**Knee Flex Torque NormHM**	Max, Min	Nm/kgm	
**Hip Flex Torque NormHM**	Max, Min	Nm/kgm	
**L5S1 Flex Torque NormHM**	Max, Min	Nm/kgm	

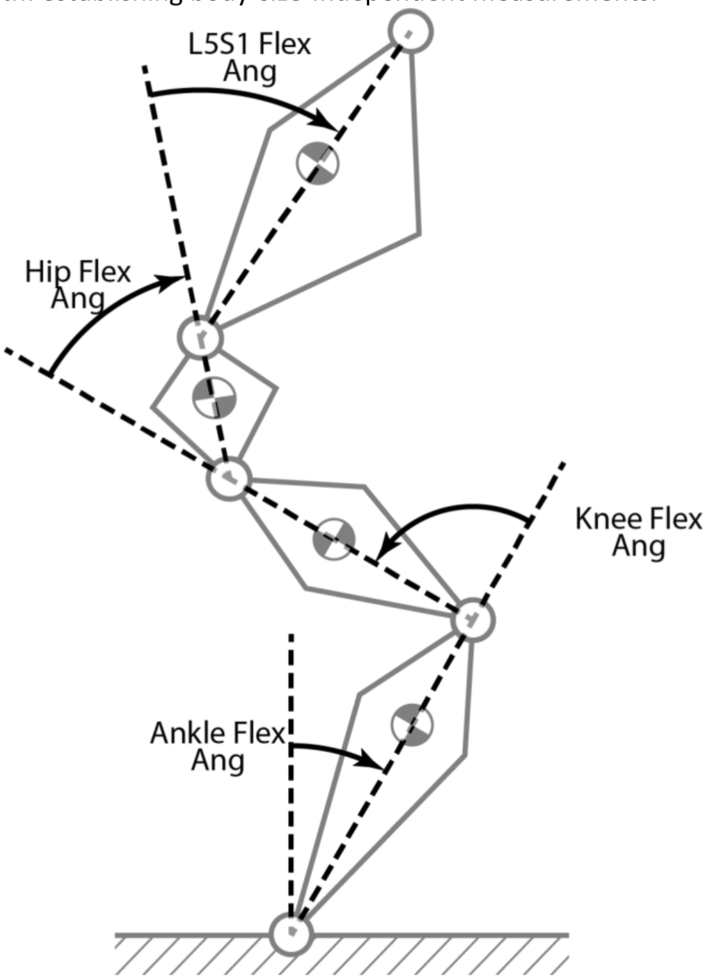

3.2 Joint Powers

ID	Variant	Units	Description
**Ankle Flex Power**	Max, Min	W	Computed from torques and angular velocities
**Knee Flex Power**	Max, Min	W	
**Hip Flex Power**	Max, Min	W	
**L5S1 Flex Power**	Max, Min	W	
**Ankle Flex Power NormHM**	Max, Min	W/kgm	Powers normalized by subject Mass x Height
**Knee Flex Power NormHM**	Max, Min	W/kgm	
**Hip Flex Power NormHM**	Max, Min	W/kgm	
**L5S1 Flex Power NormHM**	Max, Min	W/kgm	

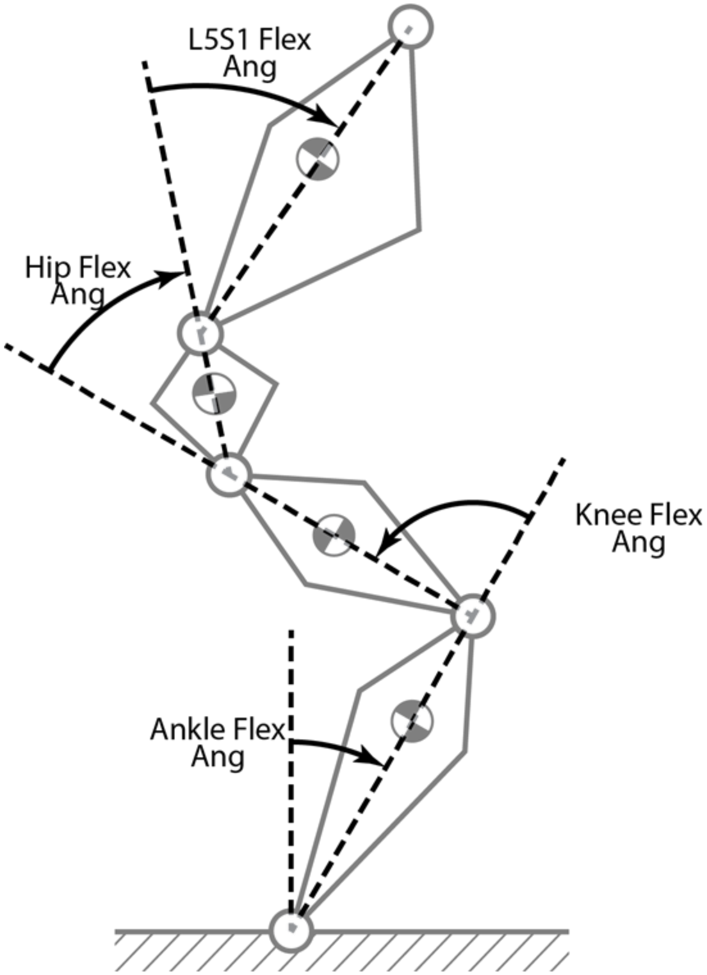

4 Quaternary Measures

ID	Variant	Units	Description
L5S1 Extensor Muscle Force	Max	N	Computed using Chaffin2006 Occupational Biomechanics planar low back model
L5S1 Lumbar Anterior Shear	Max	N	
L5S1 Lumbar Compression	Max	N	
L5S1 Sacral Anterior Shear	Max	N	
L5S1 Sacral Compression	Max	N	
L5S1 Extensor Muscle Force NormM	Max	N/kg	Forces Normalised by Mass
L5S1 Lumbar Anterior Shear NormM	Max	N/kg	
L5S1 Lumbar Compression NormM	Max	N/kg	
L5S1 Sacral Anterior Shear NormM	Max	N/kg	
L5S1 Sacral Compression NormM	Max	N/kg	

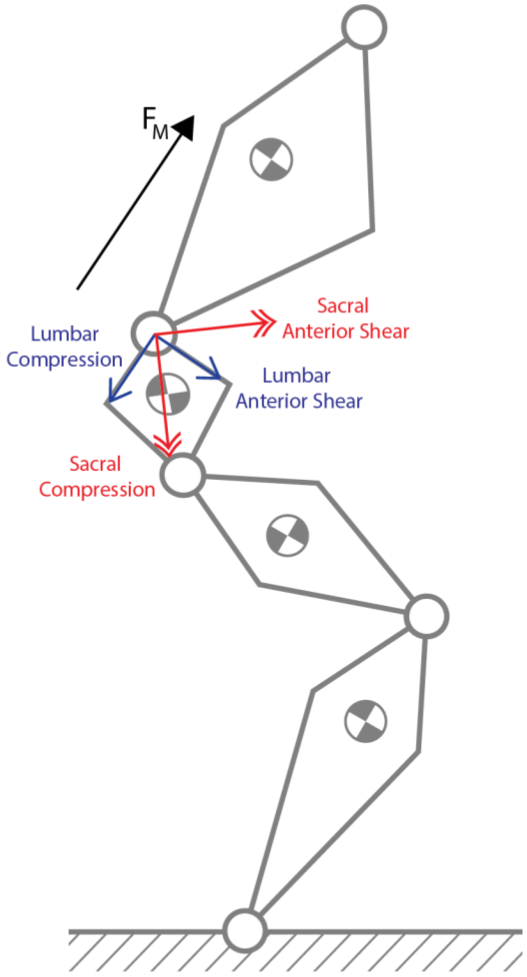

Supplemental figure 1. The plot of the variance accounted for in the original vs. permuted solution (a) and the statistical results (b)

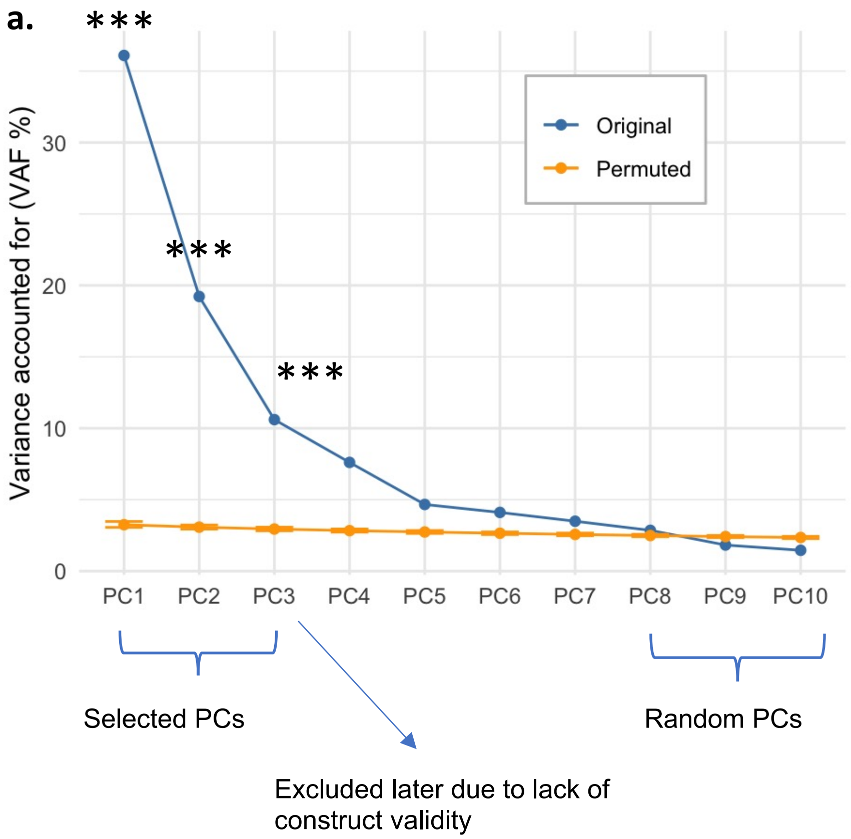

b.

	original	mean	ci_low	ci_high	pvalue	adj.p.value
PC1	0.3609539	0.0325221	0.0307366	0.0347297	0.000999	0.0024975
PC2	0.1922242	0.0307965	0.0294421	0.0323887	0.000999	0.0024975
PC3	0.1060363	0.0294858	0.0282637	0.0309776	0.000999	0.0024975
PC4	0.0760941	0.0284033	0.0272617	0.0295532	0.000999	0.0024975
PC5	0.0466445	0.0274164	0.0263554	0.0284930	0.000999	0.0024975
PC6	0.0410726	0.0265177	0.0255563	0.0274857	0.000999	0.0024975
PC7	0.0349176	0.0256934	0.0248020	0.0266991	0.000999	0.0024975
PC8	0.0285397	0.0249209	0.0240764	0.0258052	0.000999	0.0024975
PC9	0.0182560	0.0241698	0.0233976	0.0250391	1.000000	1.0000000
PC10	0.0145684	0.0234939	0.0227243	0.0243133	1.000000	1.0000000

Supplemental Table 1. Pattern matching statistic results of the original vs bootstrapped first 3 PCs

PC	cc_index	r_correlation	rmse	s_index
1	0.9972143	0.9944000	0.0450323	0.9824260
2	0.9957557	0.9952713	0.0420380	0.9534910
3	0.9908463	0.9914017	0.0450613	0.9350533

PC	cc_index	r_correlation	rmse	s_index
1	0.9911325	0.9806975	0.0268475	0.9592325
2	0.9909950	0.9897900	0.0275475	0.9112850
3	0.9625425	0.9657850	0.0252475	0.8784750

PC	cc_index	r_correlation	rmse	s_index
1	0.9990525	0.9981525	0.0818100	0.9956000
2	0.9982525	0.9980000	0.0650775	0.9849000
3	0.9971525	0.9972525	0.0911400	0.9763575

Supplemental figure 2. Full loadings of the first three principal components of the NLPCA solution with confidence intervals.

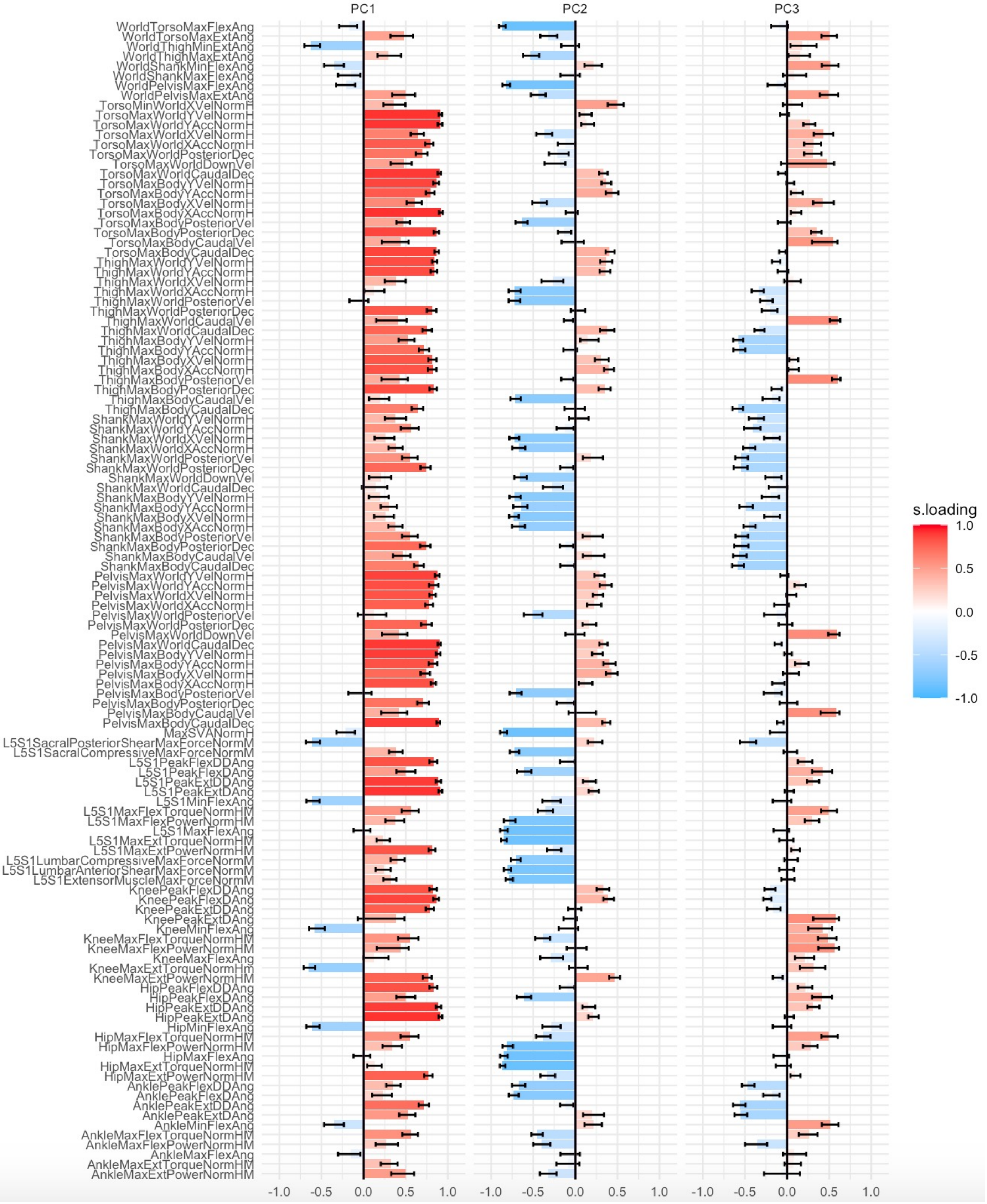

Supplemental figure 3. PC3 is a diffuse complex construct.

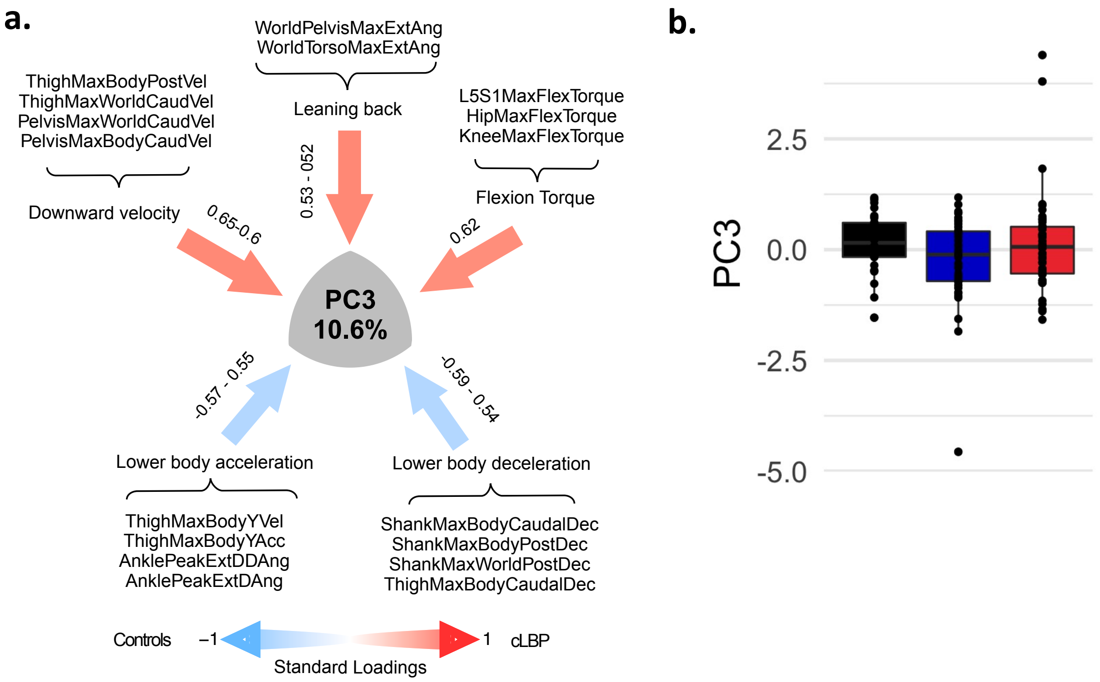

Supplemental figure 3. PC3 was excluded from the main line of interpretation due to inability to identify a cohesive pattern of loadings. a. Kinematic, dynamic as well as kinetic variables have loaded in PC3 into separate STS movement components that do not link or group into a strategy as do PC1 and PC2. b. There were no significant differences in PC3 scores between groups. c. PC3 scores vs. VASback linear regression model plot.

Supplemental figure 4. PC4- PC6 loadings plots.

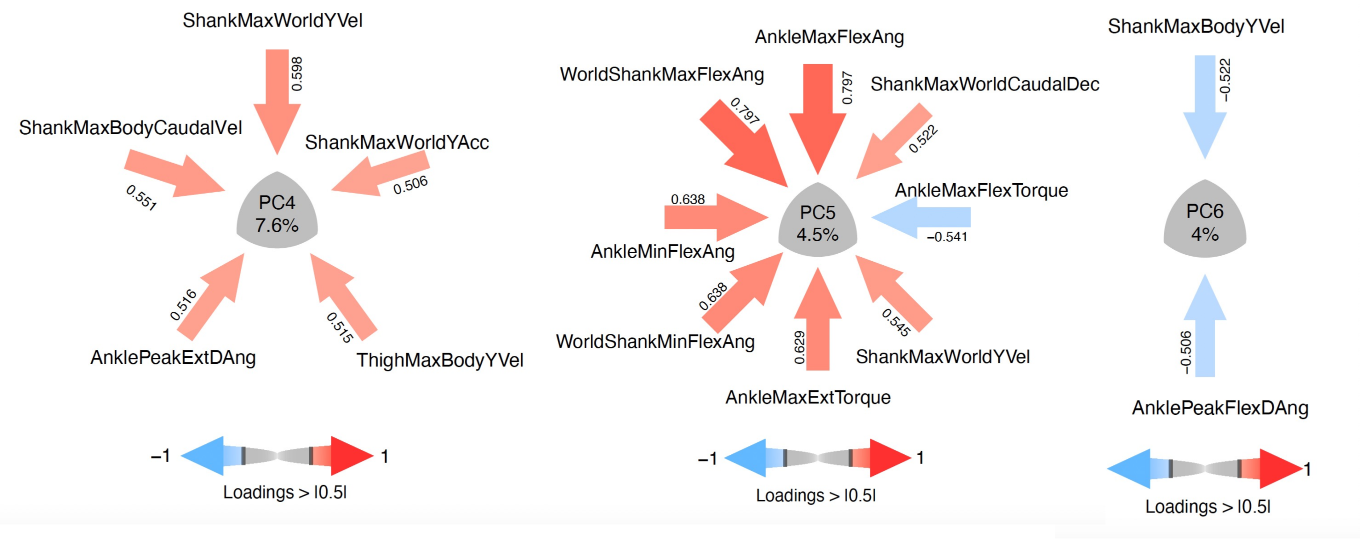

Supplemental figure 4. The construct PC4-PC7 were excluded from the main analysis following Kaiser and Scree plot rules. However, based on the permutation test PC4- PC7 have significant loadings/information. P7 did not contain any loadings above 0.5 and was excluded from further investigation. We, therefore, report the loadings in the supplementary materials for PC4- PC6, which together account for additional 16.1% variance in the the data and capture the information related to participants ankle biomechanics.

Abbreviations:

Acc – acceleration
Ang – angle
BMI – body mass index
D – joint velocity
DD – joint acceleration
Deg – degrees
CC_ index – Congruence coefficient
CI – confidence Interval
cLBP – chronic low back pain
CNS – central nervous system
Ext – extension
Flex – flexion
H- height
LBP – low back pain
M – mass
m/s – meters per second
Max – maximum
Min – minimum
NLPCA – non-linear principal component analysis
Norm – normalized
NS-LBP – non-specific low back pain
ODI – Oswestry disability index
PC – principal component
RMSE – root mean squared error
S. loading – standardized loading
SD-LBP – spinal deformity induced low back pain
S_index – similarity index
STS – sit to stand
SVA – sagittal vertical axis
T – time
VAF – variance accounted for
VAS – visual analogue scale
Vel – velocity
